# Supplementary material for: Preserving friendships in school contacts: An algorithm to construct synthetic temporal networks for epidemic modelling
Source: PLoS Comput Biol. 2024 Dec 9;20(12):e1012661. doi: 10.1371/journal.pcbi.1012661 (PMC11676524; doi:10.1371/journal.pcbi.1012661)
Supplement: S1 Text — (PDF) [file pcbi.1012661.s001.pdf]

Supplementary Information to: Preserving friendships in school contacts: an algorithm to construct synthetic temporal networks for epidemic modelling

Lucille Calmon[1], Elisabetta Colosi[1,+], Giulia Bassignana[1], Alain Barrat[†2] and Vittoria Colizza[†*1,3]

[1]Sorbonne Université, INSERM, Pierre-Louis Institute of Epidemiology and Public Health (IPLESP), Paris, France

[2]Aix Marseille Univ, Université de Toulon, CNRS, CPT, Turing Center for Living Systems, Marseille, France

[3]Department of Biology, Georgetown University, Washington, District of Columbia, USA

+Current address: Bocconi University, Dondena Centre for Research on Social Dynamics and Public Policy, Milan, Italy

†These authors contributed equally: Alain Barrat and Vittoria Colizza

∗Corresponding author: Vittoria Colizza (vittoria.colizza@inserm.fr)

November 26, 2024

Contents

A Repeated contacts as friendships between students **2**

B Synthetic contact generation **4**

B.1 Friendship-based approach 4

B.1.1 Algorithm 4

B.1.2 Optimization of the parameters 6

B.1.3 Sensitivity on the distance measure optimised 7
B.2 Class-mixing-based approach 10

C Transmission model **12**
C.1 Disease progression model and parameters 12
C.2 Transmission probability 13
C.3 Calibration of the transmission rate 14

D Infection pathways **15**
D.1 Infection pathways between students 15
D.2 Infection pathways between classes 15

E Individual impacts of f and p_{tr} **16**

F Additional figures **22**

A Repeated contacts as friendships between students

In addition to the deployment of sensors to record contacts, a fraction of the students (134 students) filled out a survey where they named their friends [1]. We compared the resulting directed network of friendships to the daily networks of contact links to measure the probability that a repeated contact link occurs between a pair of declared friends.

In the following, empirical daily networks of contacts and the directed static friendship network are restricted to nodes present in both networks (a total of 133 nodes). Any pair of nodes (i, j) can be classified as follows: (i, j) are never in contact, this is a "no contact link"; (i, j) are in contact on one day of the deployment only, this is an "unrepeated contact link"; and finally a contact link between (i, j) is observed on at least two days of the deployment (not necessarily consecutive), in which case (i, j) is a "repeated contact link". Moreover, each pair of students can be characterised by their friendship status: "no friendship" if neither i nor j reports a friendship with j nor i, "friendship" if i and/or j reports a friendship with j and/or i (i.e., at least one of the individual declares a friendship with the other) and finally (i, j) becomes a "reciprocal friendship" when both i and j declare a friendship with each other. Note that reciprocal friendships are also friendships in this classification.

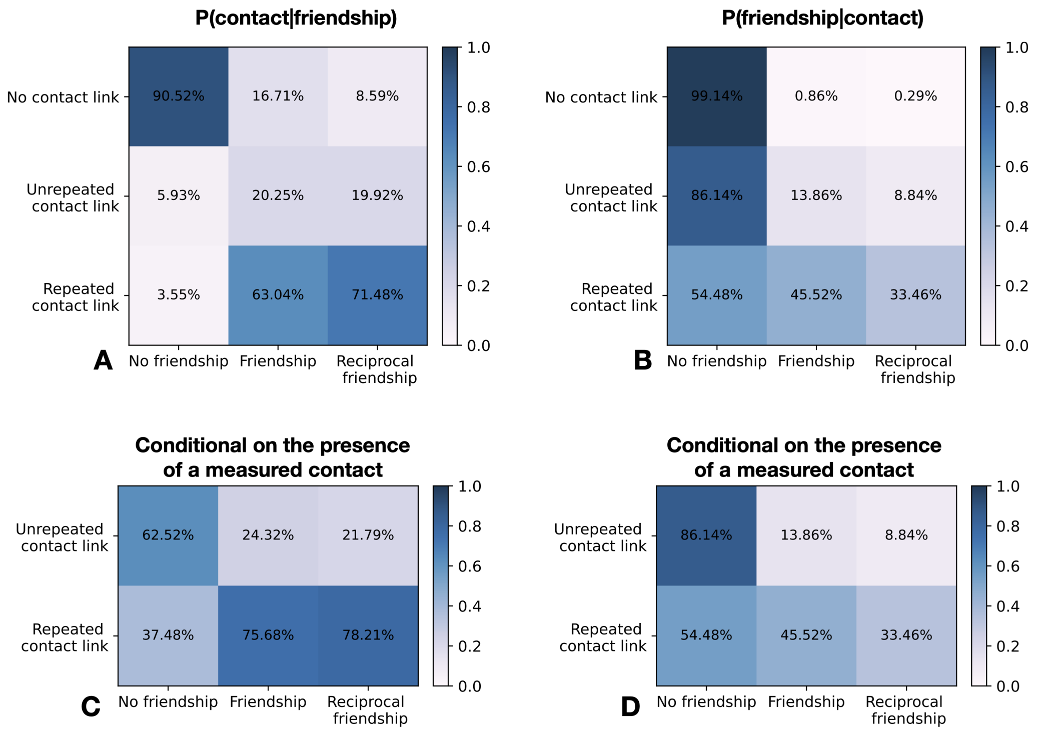

Figure A: Panel A shows the probability of a contact link to be absent, unrepeated or repeated, conditional on the friendship status of the link. Instead panel B presents the probability of a pair of students to correspond to no friendship, a friendship (inc. a non-reciprocal one), and a reciprocal friendship, conditional on the type of contact observed for that link (absent, unrepeated or repeated). Panels C and D shows the same conditional probabilities as panels A and B, with the additional condition that a contact is observed between the pairs. Note that B and D are identical.

Conditional probabilities of the type of contact link given the links' friendship status shown in Fig. A indicate that pairs of students with no declared friendship are most likely to not be in contact at all during the deployment (90.52%) (panel A). If they are in contact, they are more likely (62.52%) to be in unrepeated contacts (panel C). Instead, pairs of students who declared a friendship (reciprocal or not) are most likely to correspond to repeated contact links (63.04% of all friendship pairs, and 75.68% of those in contacts) (panels A and C). Inversely, panel B and D show that unrepeated contact links mostly correspond to pairs of students with no friendship declared (86.14% of unrepeated contact links). We therefore conclude that the presence of a friendship among students is likely to lead to the emergence of repeated links in the recorded daily contact networks. When working with the full set of contacts, regardless of declared friendship status, we call for simplicity repeated links "friendship links", and unrepeated links "casual links".

B Synthetic contact generation

B.1 Friendship-based approach

B.1.1 Algorithm

We provide here a detailed description of the friendship-based approach, on a base day d. For each pair of classes A and B (including $A = B$), obtain from the data set:

- Number of contact links N^d_{AB} occurring on d between classes A and B (i.e. number of links (i, j) with i in class A and j in class B or vice-versa on the day d). if $A = B$, N^d_{AA} is the number of contact links among the students of class A on day d.
- List of contact links between individuals of classes A and B that occur on day d, $\{(i, j)\}^d_{AB}$, as well as the list of their weights $W^d_{AB} = \{w_{ij}\}$ and timelines $TL^d_{AB} = \{tl_{ij}\}$. If $A = B$ these are all links among students of the class A.
- All friendship links of day d (links repeated on d and at least $d' \neq d$) between individuals of classes A and B in a dictionary $\mathbf{Fr}^d_{AB}[(i, j)] = \{weights : \{w\}_{ij},\ timelines : \{tl\}_{ij}\}$, where $\{w\}_{ij}$ is the list of weights of (i, j), and $\{tl\}_{ij}$ the list of timelines of (i, j) on all days the friendship link is realised. If $A = B$ these are all friendship links among students of the class A.

Create a synthetic daily contact network between classes A and B as follows (and within class when $A = B$).

- For each friendship link (i, j) in the keys of the dictionary $\mathbf{Fr}^d_{AB}$: include it in the synthetic network with probability f. If included, build a timeline as follows.
  - Draw a target weight uniformly between w_{min} and w_{max}, the minimum and maximum weights observed on different days for the included link (i, j) ($w_{min} = min(\{w\}_{ij})$, $w_{max} = max(\{w\}_{ij})$).
  - Take the timeline in $\{tl\}_{ij}$ corresponding to w_{min}. Randomly add to it timestamps from $\{tl\}_{ij}$ until the target weight selected in the previous step is reached.
- The synthetic network between classes A and B now contains approximately $f * |\mathbf{Fr}^d_{AB}|$ interactions

where $|\mathbf{Fr}^d_{AB}|$ is the number of friendship links of the class or pair of classes that also occur on day d. Add subsequent contact links by repeating the next three bullet points until the synthetic network contains N^d_{AB} contact links.

  - With probability p_{tr}: add a link while accounting for its effect on transitivity as follows. If the transitivity [2] (fraction of closed triangles, i.e. of structures $\{(i,j),(j,k),(k,i)\}$ among all possible connected triads, i.e., such that at least $\{(i,j),(j,k)\}$ exist) in the network of synthetic contact links constructed so far is higher than in the empirical network of contact links between classes A and B or within class $A = B$: add a link that necessarily decreases the transitivity (by opening a triangle). If it is too low instead, add a link that necessarily increase transitivity by closing a triangle. Add the contact link from the subset of empirical contact links $\{(i,j)\}^d_{AB}$ that have the correct effect on transitivity with probability p, or else, add a contact link that has the correct effect on transitivity picked randomly between any two individuals in classes A and B (probability $1-p$).
  - Else, with probability $1-p_{tr}$: add a contact link regardless of the effect on transitivity from empirically observed links in $\{(i,j)\}^d_{AB}$ (probability p) or between any pair of students in classes A and B (probability $1-p$).
  - Associate to the link added in the synthetic contact network a weight and (matching) timeline drawn randomly from $W^d_{AB} = \{w_{ij}\}$ and $TL^d_{AB} = \{tl_{ij}\}$.

- Once the correct number of links between classes A and B (or within class $A = B$), has been added (namely N^d_{AB}), we proceed to control that the total time spent in contact between classes A and B (or within class $A = B$) is within a certain tolerance ($TOL = 0.1$) from the observed total time in contact between the same pair of classes (or within the same class). While the total time in contact in the synthetic contact network ($T^d_{syn,AB} = \sum_{ij} w_{ij}$ over all links (i,j) between classes A and B or within class $A = B$) is higher than observed for d for that pair of class, remove timestamps randomly from longer timelines preferably, repeating the following steps.

  - Each contact link (i,j) is associated a probability $p^d_{ij,AB} = w_{ij}/T^d_{syn,AB}$ where w_{ij} is the weight for that contact link.

– Pick a link according to the discrete probability distribution $\{p^d_{ij,AB}\}$, then pick a timestamp randomly in the timeline of the link picked, and remove it.

– Repeat.

- Instead, while the total time in contact ($T^d_{syn,AB}$) is lower than observed in the day copied for that pair of class, add timestamps randomly to longer timelines preferably, repeating the following steps.

  – Each contact link (i,j) is associated a probability $p^d_{ij,AB} = w_{ij}/T^d_{syn,AB}$ where w_{ij} is the weight for that contact link.

  – Pick a link according to the discrete probability distribution $\{p^d_{ij,AB}\}$, then pick a timestamp ts randomly in the timeline of the link picked. If $ts + 20$ sec, i.e. the following timestamp in steps given by the resolution, is not already in the timeline of the link, add it.

  – Repeat.

B.1.2 Optimization of the parameters

The parameters f, p and p_{tr} are optimised as follows. For triplets of parameters (f, p, p_{tr}) each ranging between 0 and 1, we generate 10 synthetic realisations (indexed by $n = 1, 2, ..., 10$) based on each day in the data (indexed by $d = 2, 3, 4, 5$), each denoted by $G_{fr,n,d}(f, p, p_{tr})$. For each realisation n, the local cosine similarity (Eq. 1 in the main text) computes for each student and each pair of base days the similarity in contact links. The synthetic distribution of local cosine similarities, denoted by $LCS_{pdf,fr}(f, p, p_{tr})$ is computed over all students, all iterations n and all pairs of days. Simultaneously, the local cosine similarity can be measured between the contacts of each student, for each pair of days of the deployment. The resulting distribution (over students and pairs of days), denoted by $LCS_{pdf,emp}$ is compared to $LCS_{pdf,fr}(f, p, p_{tr})$ for each triplet of parameters with the Jensen-Shannon distance [3].

The Jensen-Shannon distance is defined as the square root of the Jensen-Shannon divergence [4], itself given by a symmetrisation of the Kullback-Leibler divergence [5]. The Kullback-Leibler divergence is

defined for two probability distributions P and Q as

$$D_{KL}(P,Q) = \sum_x P(x) \ln \frac{P(x)}{Q(x)}. \tag{1}$$

The Kullback-Leibler divergence measures the difference from the distribution $P(x)$ to the baseline $Q(x)$. It can be interpreted as the relative entropy of $P(x)$ with respect to $Q(x)$. This measure is however asymmetric, and unbounded.

The Jensen-Shannon divergence [4] is instead given by

$$D_{JS}(P,Q) = \frac{1}{2}(D_{KL}(P,M) + D_{KL}(Q,M)) \tag{2}$$

where $M(x) = \frac{1}{2}(P(x)+Q(x))$ acts as a single baseline for both distributions. This divergence measure has the advantage of being symmetric, and bounded. The Jensen-Shannon distance, given by

$$d_{JS}(P,Q) = \sqrt{D_{JS}(P,Q)}, \tag{3}$$

is formally a metric [6]. We therefore use $d_{JS}(P,Q)$ in our analysis to measure the distance between $LCS_{pdf,emp}$ and $LCS_{pdf,fr}(f,p,p_{tr})$ for each triplet of parameters.

As shown in Fig. B, the parameter f has the most impact on the similarity distribution. This is due to the fact that this parameter controls the inclusion of repeated interactions with their observed characteristics. Instead, the parameters p and p_{tr} have less impact on the similarity. The triplet of values associated to the minimum distance is given by $f = 0.8$, $p = 0.4$ and $p_{tr} = 0.75$. Note that several other pairs (p, p_{tr}) lead to similar performances, with Jensen-Shannon distance differing only at three significant figures.

B.1.3 Sensitivity on the distance measure optimised

We considered the following three additional distances to measure the similarity between $LCS_{pdf,emp}$ and $LCS_{pdf,fr}(f,p,p_{tr})$.

The first alternative measure we considered is the total variation distance. This distance can be defined

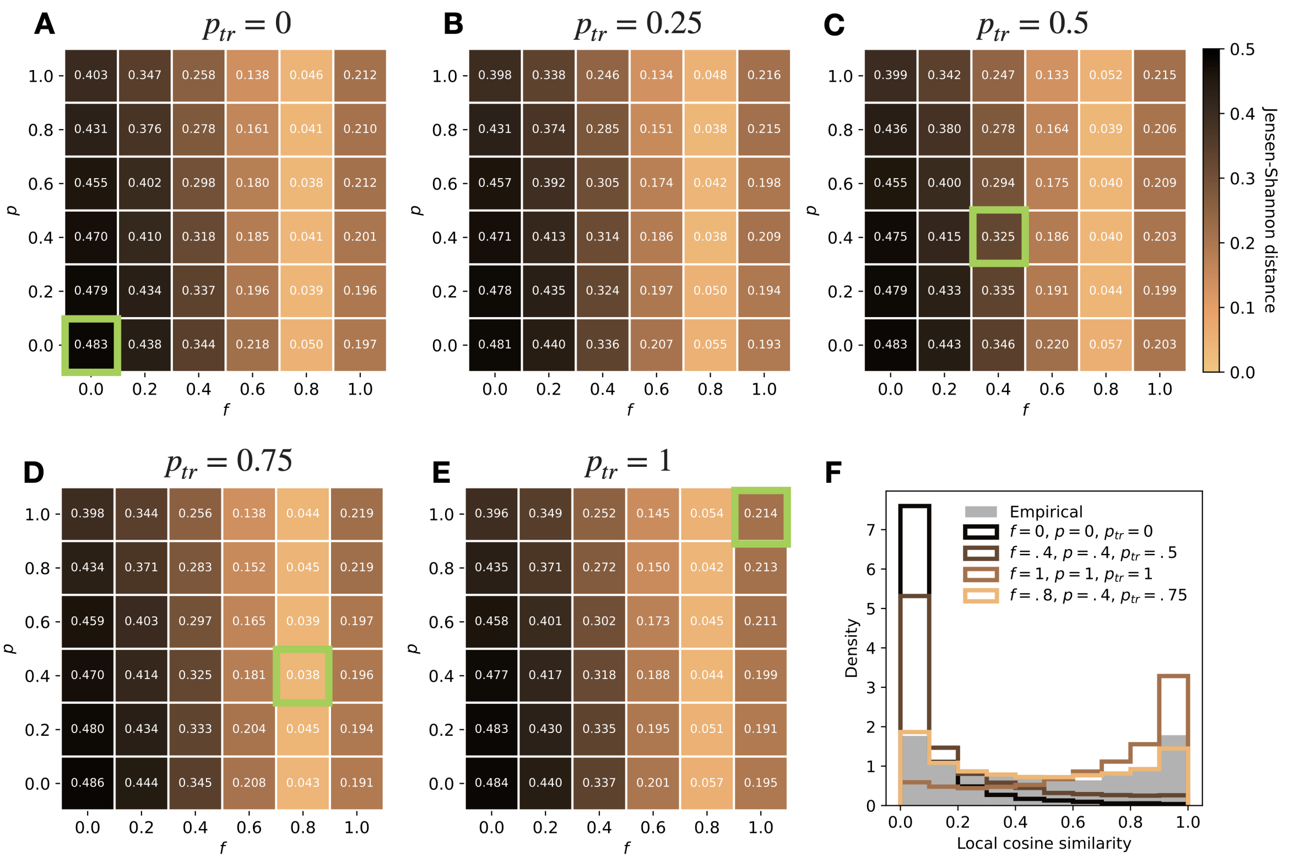

Figure B: Values of the Jensen-Shannon distance between distributions of local cosine similarities obtained from empirical contacts ($LCS_{pdf,emp}$) and contacts obtained from the friendship-based approach ($LCS_{pdf,fr}(f, p, p_{tr})$) generated with varying values of f, p (x and y axis) and p_{tr} ($p_{tr} = 0$, 0.25, 0.5, 0.75 and 1 in panels A, B, C, D and E) are shown in panels A to E. Lowest values of the Jensen-Shannon distance correspond to triplets of parameters leading to distributions of local cosine-similarities most similar to $LCS_{pdf,emp}$. Panel F instead shows the distributions $LCS_{pdf,emp}$ and $LCS_{pdf,fr}(f, p, p_{tr})$ obtained with four different triplets of parameters, highlighted in green in corresponding panels. Values shown in panels A to E are rounded to three decimals.

as [7]

$$d_{TVD}(P, Q) = \frac{1}{2} \sum_x |P(x) - Q(x)|. \tag{4}$$

The value of the total variation distance for different triplets of parameters is reported in Fig. C. This distance is minimal for $f = 0.8$, $p = 0.4$, $p_{tr} = 0.75$ as was the case with the Jensen-Shannon distance.

The Hellinger distance is an alternative measure of similarity between two probability distributions, defined as [8]

$$d_H(P, Q) = \sqrt{2\left(1 - \sum_x \sqrt{P(x)Q(x)}\right)}. \tag{5}$$

Its value for different triplets of parameters is reported in Fig. D. This distance again is minimal for

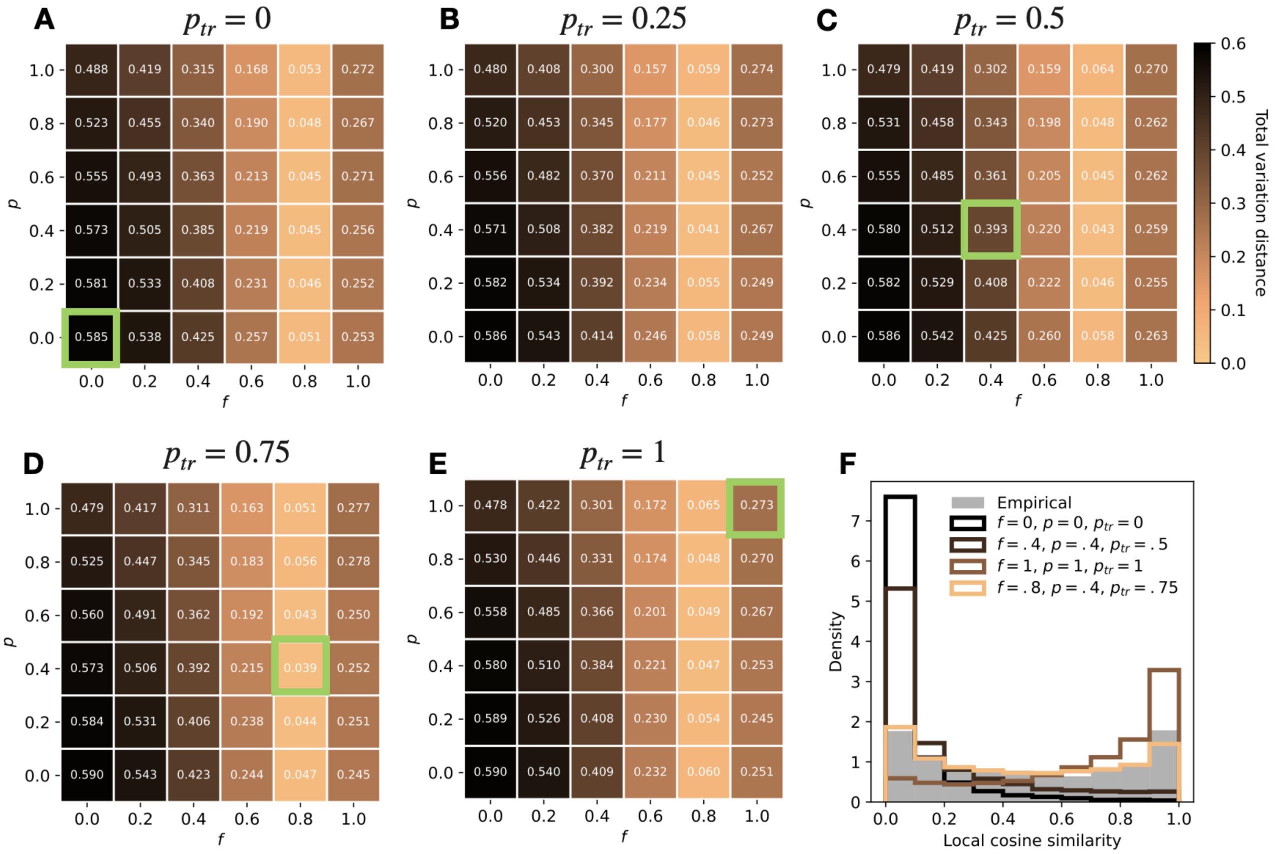

Figure C: Values of the total variation distance between distributions of local cosine similarities obtained from empirical contacts ($LCS_{pdf,emp}$) and contacts obtained from the friendship-based approach ($LCS_{pdf,fr}(f,p,p_{tr})$) generated with varying values of f, p (x and y axis) and p_{tr} ($p_{tr} = 0$, 0.25, 0.5, 0.75 and 1 in panels A, B, C, D and E) are shown in panels A to E. Panel F instead shows the distributions $LCS_{pdf,emp}$ and $LCS_{pdf,fr}(f,p,p_{tr})$ obtained with four different triplets of parameters, highlighted in green in corresponding panels. Values shown in panels A to E are rounded to three decimals.

$f = 0.8$, $p = 0.4$, $p_{tr} = 0.75$.

Finally, we additionally considered the Euclidean distance, defined as

$$d(P,Q) = \sqrt{\sum_x (P(x) - Q(x))^2}. \tag{6}$$

The Euclidean distance is shown for different triplets of parameters in Fig. E. This distance is not minimal for $f = 0.8$, $p = 0.4$, $p_{tr} = 0.75$. Instead, its minimum corresponds to the parameter triplet $f = 0.8$, $p = 0.8$, $p_{tr} = 0.25$. However, the value of the Euclidean distance for the triplets optimising the remaining of the distances is very close to the minimum (0.387930 versus 0.353763 at the minimum).

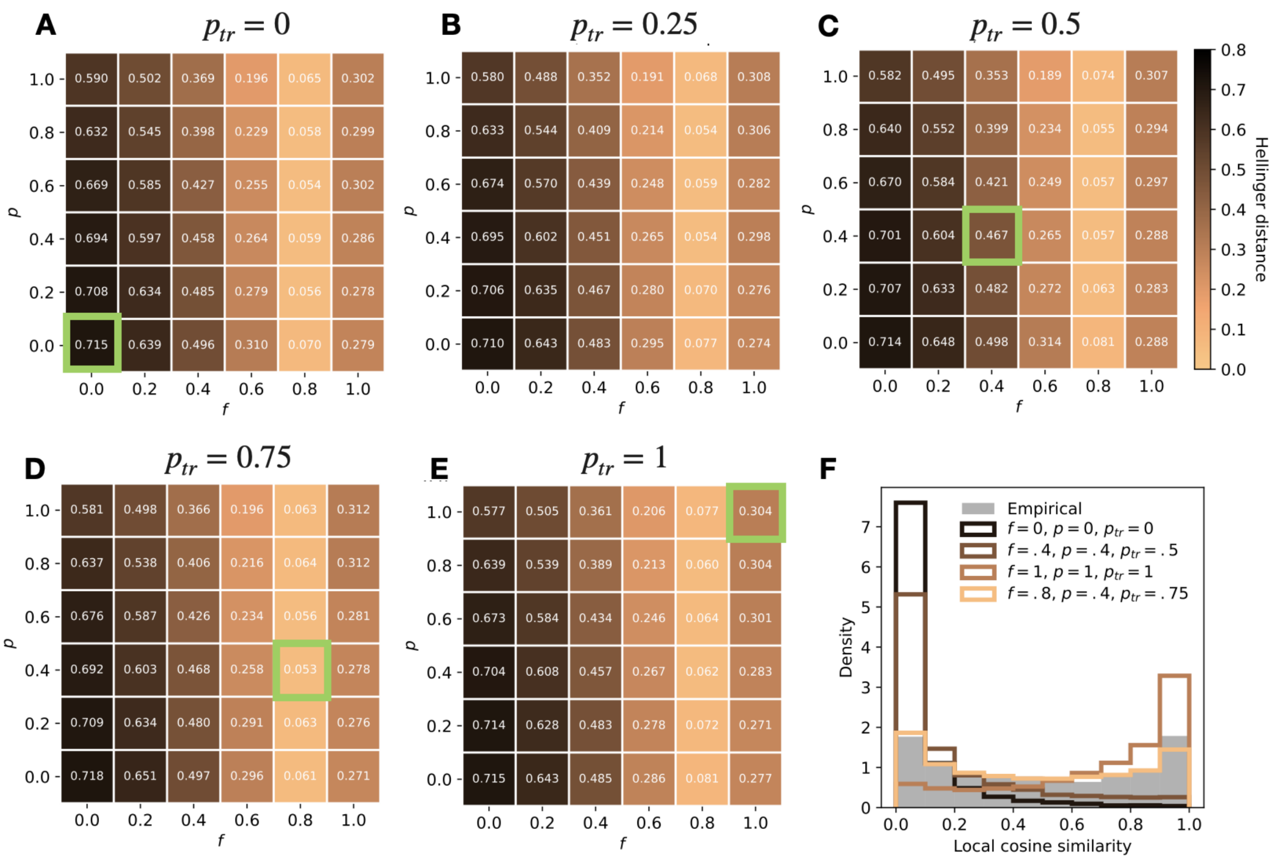

Figure D: Values of the Hellinger distance between distributions of local cosine similarities obtained from empirical contacts ($LCS_{pdf,emp}$) and contacts obtained from the friendship-based approach ($LCS_{pdf,fr}(f, p, p_{tr})$) generated with varying values of f, p (x and y axis) and p_{tr} ($p_{tr} = 0$, 0.25, 0.5, 0.75 and 1 in panels A, B, C, D and E) are shown in panels A to E. Panel F instead shows the distributions $LCS_{pdf,emp}$ and $LCS_{pdf,fr}(f, p, p_{tr})$ obtained with four different triplets of parameters, highlighted in green in corresponding panels. Values shown in panels A to E are rounded to three decimals.

B.2 Class-mixing-based approach

We provide here a detailed description of the class-mixing-based approach, on a base day d. For each pair of classes A and B (including each single class $A = B$), obtain from the data set:

- Number of interactions N^d_{AB} between A and B over the day d.
- List of the weights $W^d_{AB} = \{w_{ij}\}$ and timelines $TL^d_{AB} = \{tl_{ij}\}$ of the contact links occurring on d between classes A and B or within class $A = B$.

Create a synthetic daily contact network between classes A and B, repeating the steps below until the synthetic network between classes A and B contains N^d_{AB} contact links.

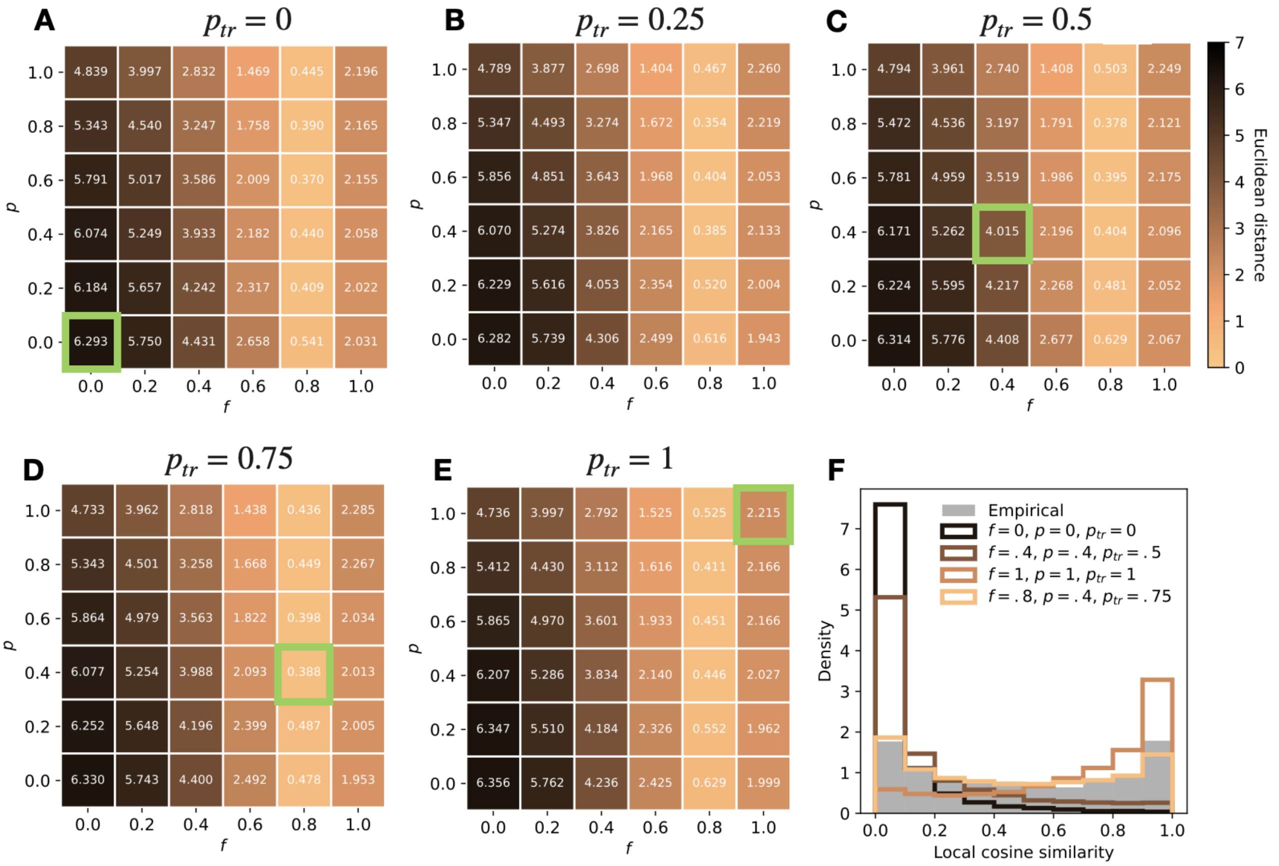

Figure E: Values of the Euclidean distance between distributions of local cosine similarities obtained from empirical contacts ($LCS_{pdf,emp}$) and contacts obtained from the friendship-based approach ($LCS_{pdf,fr}(f, p, p_{tr})$) generated with varying values of f, p (x and y axis) and p_{tr} ($p_{tr} = 0$, 0.25, 0.5, 0.75 and 1 in panels A, B, C, D and E) are shown in panels A to E. Panel F instead shows the distributions $LCS_{pdf,emp}$ and $LCS_{pdf,fr}(f, p, p_{tr})$ obtained with four different triplets of parameters, highlighted in green in corresponding panels. Values shown in panels A to E are rounded to three decimals.

- Add a link between any two individuals (i in A and j in B or vice versa) picked randomly among the students of each class.
- Associate the link a weight and (matching) timeline drawn randomly from $W^d_{AB} = \{w_{ij}\}$ and $TL^d_{AB} = \{tl_{ij}\}$.

Repeat this process until all pairs of classes A, B (including all classes $A = B$) have been considered. Collating all contact links and their characteristics (weight and timelines) provides a synthetic daily contact network based on day d.

C Transmission model

The transmission model of SARS-CoV-2 we use was initially developed and introduced in [9], then subsequently reused in [10, 11]. We parameterised the model to reproduce the disease progression of the Omicron variant [10], and calibrated the transmission rate β to ensure an effective reproductive number $R = 1.5$ in the resulting outbreaks.

C.1 Disease progression model and parameters

The progression of the disease is encoded by the model schematically represented in Fig. F. Upon infection, an individual becomes first exposed (E), but cannot transmit the disease. After this latent phase, lasting τ_E, the individual enters the pre-symptomatic phase (I_p) during which they are infectious prior to showing clinical signs. This phase lasts τ_p, after which the individual can remain without symptom (in the subclinical phase, I_{sc}) with probability p_{sc} or instead develop symptoms and enter the clinical phase (I_c) with probability $(1 - p_{sc})$. Either phases last τ_I after which the individual becomes immune and cannot transmit the disease anymore ($R+$, in which PCR tests can still detect the virus in upper respiratory tracks, then R). The duration of each disease stage are modelled by Gamma distributions with parameters informed by the literature, and given in Table 1.

Disease stage	Shape	Mean (days)	Transition rate to the next stage (days^{-1})
Exposed (E)	3	3.5	$\epsilon = 0.285$
Prodromic (I_p)	1	1.8	$\mu_p = 0.555$
Clinical (I_c and I_{sc})	6	5	$\mu = 0.2$
Immune testing positive ($R+$)	2	24.5	$\mu_{R+} = 0.04$

Table 1: Parameters informing the Gamma distributions characterising the duration of each disease stage, and the transition between stages.

The model described in Fig. F is additionally stratified to describe the disease progression in vaccinated individuals.

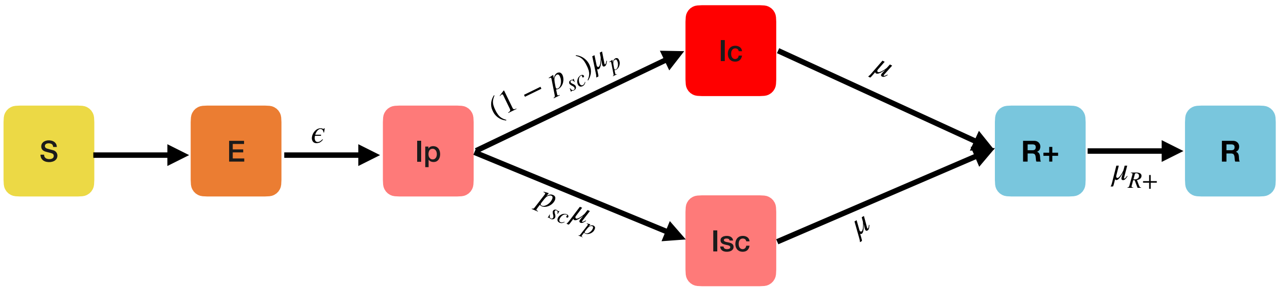

Figure F: Structure and parameters of the transmission model used to describe the spread of SARS-CoV-2 in a school population. Adapted from [9].

The parameters of the model and their values are described in Table 2.

Parameter	Description	Value
p_{sc}	Probability of subclinical infection	0.8
β	Transmissibility per contact per unit time	Calibrated to reproduce $R = 1.5$
r_β	Relative transmissibility in pre-symptomatic and subclinical stages	0.55
σ_S	Relative susceptibility to infection conferred by previous infection	0.81
VE_{inf}	Vaccine effectiveness against infection	0.5
VE_{trans}	Vaccine effectiveness against transmission	0.2

Table 2: Parameters of the transmission model.

C.2 Transmission probability

Upon a contact between a pair of individuals in (S) and any of the infectious compartments (I_p, I_{sc} and I_c), disease transmission occurs with probability given by

$$p = \beta * \Delta t * w * r_\beta * \sigma_S \tag{7}$$

where $\Delta t = 15$ minutes, w is the fraction of that time spent in contact, r_β is the relative transmissibility (dependent on the infectious status of the infectious individual), σ_S is the relative susceptibility (dependent on previous infection) and β is the transmission rate.

Depending on vaccination status of the pair in contact, p is further rescaled by (1-VE_{trans}) if the infectious individual is vaccinated and (1-VE_{inf}) if the susceptible individual is vaccinated.

C.3 Calibration of the transmission rate

The transmission rate β is calibrated as follows in order to reproduce outbreaks with effective reproductive number $R = 1.5$. For each contact sequence ct_x in the set "Friendship 4d", "Class Mixing 4d" and "Looped 4d", a total of 5000 simulations are ran. Simulations are initialised with a single infectious seed, and all possible seeds are covered homogeneously by the 5000 simulations. This process is repeated for a range of values of transmissibility β. For each transmissibility β and contact sequence ct_x, the reproductive number $R(\beta, ct_x)$ is computed as the ratio of second generation infections to first generation infections averaged over all simulations.

The relationship between R and β can be well approximated by the relation

$$R^{fit}(\beta) = a(1 - e^{-b\beta}). \tag{8}$$

For each contact sequence ct_x, we fit the coefficients a and b to the measured $R(\beta, ct_x)$. By inverting the fitted relation, we obtain the (contact sequence dependent) value of $\beta(ct_x)$ that corresponds to $R^{fit}(\beta) = 1.5$. Fitted curves are shown in Fig. G.

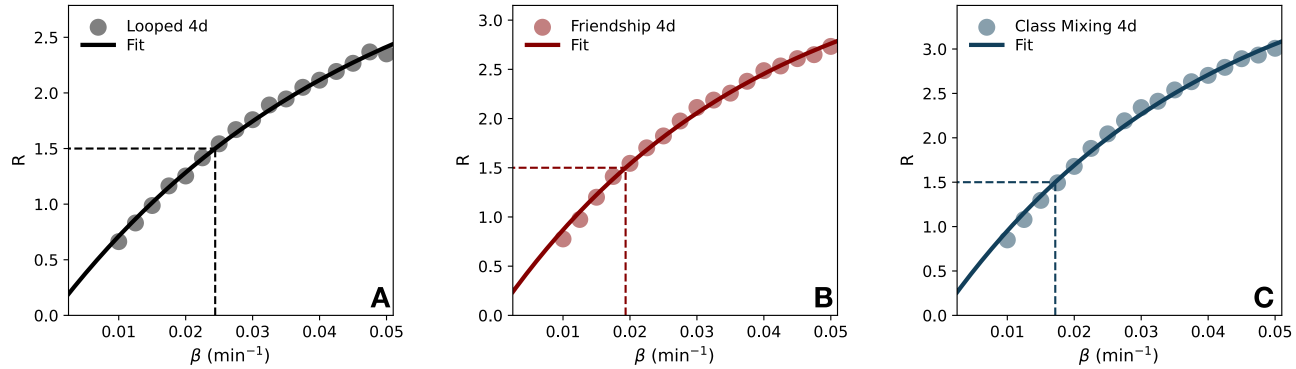

Figure G: Effective reproductive numbers obtained for each contact sequence, over a range of β. The results of the fit to Eq. (8) are shown for each contact sequence considered, together with the value of β that corresponds to $R = 1.5$. Fitted parameters are $a = 3.64$, $b = 21.75$ for "Looped 4d" contacts in panel A, $a = 3.80$, $b = 25.94$ for "Friendship 4d" contacts in panel B and $a = 4.25$, $b = 25.30$ for "Class Mixing 4d" contacts in panel C.

Values of $\beta(ct_x)$ corresponding to $R^{fit} = 1.5$ are given by β(Friendship 4d) $= 0.0193$, β(Class Mixing 4d) $= 0.0172$ and β(Looped 4d) $= 0.0244$. These values are used for remaining contact sequences built from a reduced number of based days ("3d", "2d" and "1d" sequences).

D Infection pathways

D.1 Infection pathways between students

An infection network, $\mathcal{G}_{inf}(s, ct_x)$, between students is built as follow for each given contact sequence ct_x, and a fixed seeded initial case s. Each transmission event from a student i to a student j among the $N = 150$ simulations considered is included in the infection network $\mathcal{G}_{inf}(s, ct_x)$ as a directed edge $\ell = (i, j)$ from i to j . The edge ℓ is associated a probability of occurrence given by $p_\ell(s, ct_x) = \frac{n(\ell,s,ct_x)}{N}$ where $n(\ell, s, ct_x)$ denotes the number of simulations a transmission event encoded by the edge ℓ (i.e. from i to j is observed). Note that edges are directed, and the transmission network built may include both edges (i, j) and (j, i) with different probabilities of occurrences.

The corresponding infection tree $\mathcal{T}_{inf}(s, ct_x)$ is built following the procedure detailed in [12]. In details, a weight $w_\ell(s, ct_x) = 1 - p_\ell(s, ct_x)$ is associated to each edge in $\mathcal{G}_{inf}(s, ct_x)$. The infection tree $\mathcal{T}_{inf}(s, ct_x)$ is then given by the directed minimum spanning tree obtained using the Chu–Liu / Edmonds algorithm [13, 14] implemented in the NetworkX library [15] in python [16]. The resulting structure is a directed tree rooted at the seed s, such that the sum of the cost $w_\ell(s, ct_x)$ over all (directed) edges included is minimised.

D.2 Infection pathways between classes

A similar procedure is conducted to obtain infection pathways between classes. Some care however is needed to construct the infection networks of disease progression between classes. We proceed as follows for each seed s and contact sequence ct_x.

Firstly, denote by $cl(i)$ the class of a given student i. From all observed transmission events in each simulation, retain only transmission events from student i to j if and only if $cl(j)$ has not been infected before in the simulation at the time of the transmission event. In each simulation, the retained transmission events can be viewed as a transmission chain from class to class by considering each transmission event retained as a directed edge between classes denoted $\ell = (A, B)$ where $A = cl(i)$ and $B = cl(j)$ (see panel 1 in Fig.H). We build an infection network between classes $\mathcal{G}_{inf}(s, ct_x)$ by including all such retained edges between classes, associating them a probability of occurrence given by $p_\ell(s, ct_x) = \frac{n(\ell,s,ct_x)}{N}$ where $n(\ell, s, ct_x)$ denotes the number of simulations a transmission event encoded

by the directed edge ℓ (i.e. from class A to B) is retained (see panel 2 in Fig.H). Finally an infection tree (see panel 3 in Fig.H) is built following the process described in the case of infection trees between students.

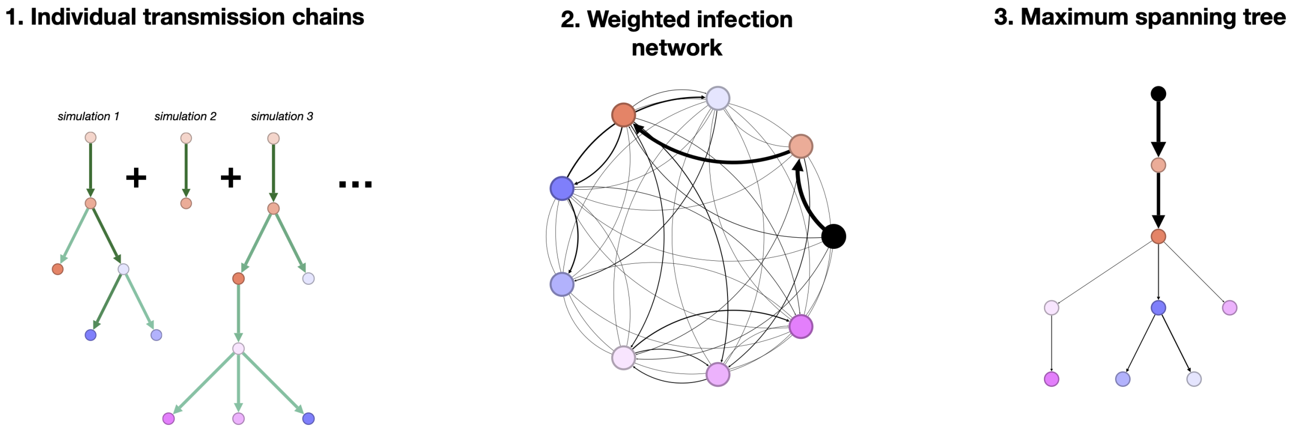

Figure H: Different simulated transmission chains between classes are shown for a given seed in panel 1 An infection network built from 135 iterations of the model with one seed is shown in panel 2 The maximum spanning tree extracted from the infection network of panel 2 is shown in panel 3. All results are obtained with "Friendship 4d" contacts. Darker edges in panel 1 correspond to transmission events occurring towards the beginning of the simulation, and lighter edges later on. Edge widths in panels 2 and 3 are proportional to the measured probability of occurrence. Nodes represent classes, and the class of the seed is highlighted in black in panels 2 and 3. Visualisations generated with Gephi [17].

E Individual impacts of f and p_{tr}

In order to tease the individual impacts of the parameters f and p_{tr}, we now compare the sequences generated with the friendship-based and class-mixing-based algorithms with two new sequences obtained from the friendship-based algorithm with either $f = 0$ (switching off the friendship mechanism) or $p_{tr} = 0$ (switching off the transitivity mechanism). In both cases, the remaining two parameters are kept to their optimised values. The fitting procedure described in Section C.3 was repeated with both contact sequences in order to obtain the values of β used for simulating the transmission model. Fitted values of β used were respectively $\beta = 0.0162$ for the contact sequence with $f = 0$, $p = 0.4$, $p_{tr} = 0.75$, and $\beta = 0.0196$ for the contact sequence with $f = 0.8$, $p = 0.4$, $p_{tr} = 0$.

Comparing the contact networks themselves (Fig. I, panel A), we find that the fraction of repeated contact links over the four base days considered is affected by the friendship mechanism ($f > 0$) but not by the transitivity preserving mechanism. Indeed, "Friendship 4d" and friendship-based contacts

with $p_{tr} = 0$ both have very similar fractions of repeated edges. Instead, setting $f = 0$ leads to a fraction of repeated links in line with that of the class-mixing-based contacts.

As shown in panel B, the transitivity itself is not strongly impacted by the transitivity mechanism. All contacts obtained with the friendship-based mechanism (regardless of the values of p_{tr}) lead to similar transitivity levels, slightly below empirical ones. This is expected. Indeed, the empirical contact networks we consider are dense, and as a result, when $f = 0.8$ and $p = 0.4$ (optimised values), we already add a high number of triangles. Instead, setting $f = 0$ and retaining $p_{tr} = 0.75$, the transitivity is only ever so slightly higher than when $f = 0.8$, $p_{tr} = 0$. However, when neither mechanisms are in place (class-mixing-based contacts), the transitivity levels decrease significantly.

The contact sequence obtained with $f = 0$ (but $p > 0$ and $p_{tr} > 0$) has local cosine similarity distribution (panel C) and global day-to-day cosine similarity (panel D) very close to the ones of the class-mixing-based contacts. This is again to be expected, as the parameter f plays a crucial role in reproducing the similarity in the contacts. Both local cosine similarity distributions and day-to-day global cosine similarities are instead not affected by the transitivity preserving mechanism.

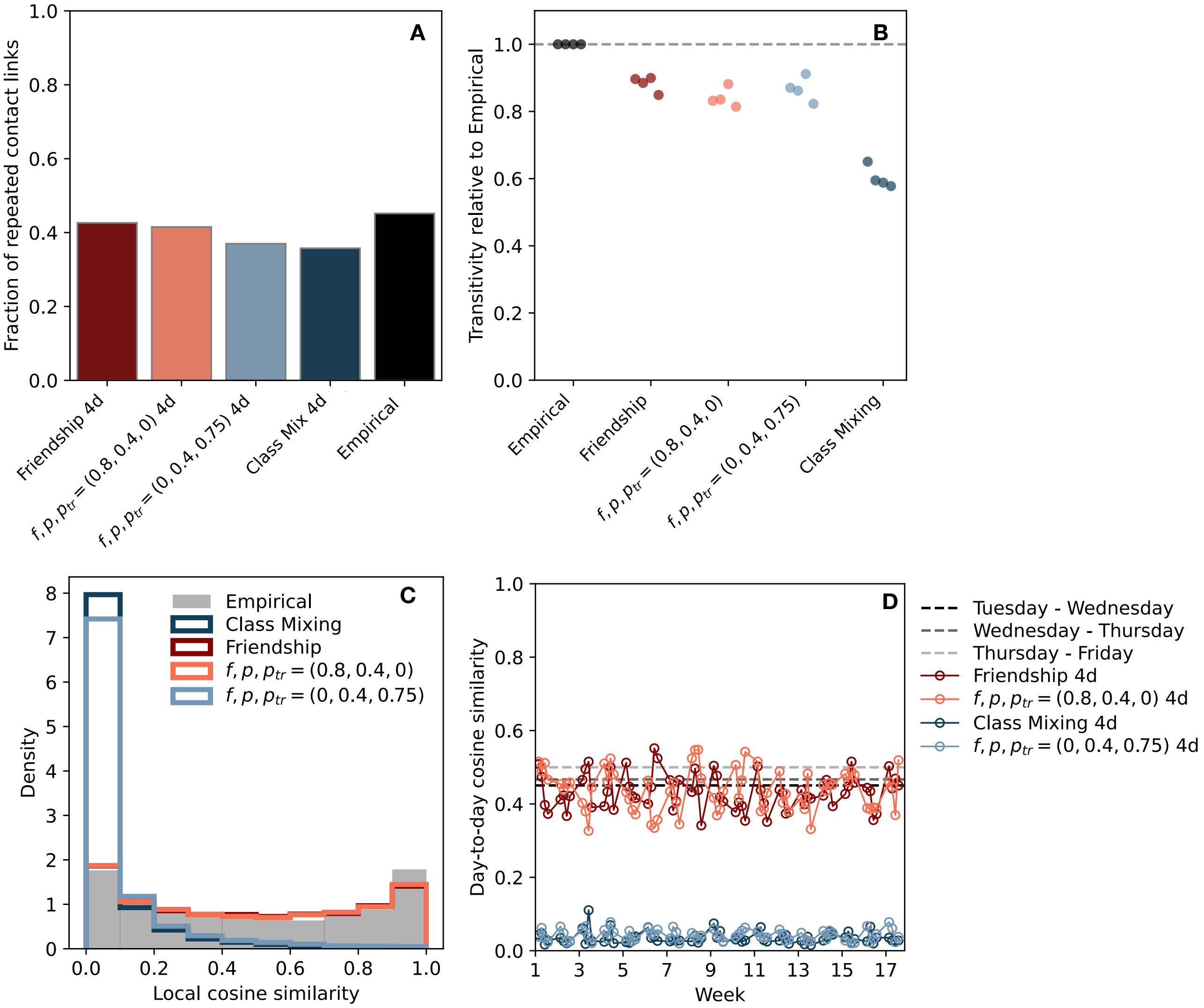

Figure I: Contacts from the friendship-based algorithm with parameter triplets $f = 0.8$, $p = 0.4$ and $p_{tr} = 0$ (transitivity mechanism turned off), $f = 0$, $p = 0.4$ and $p_{tr} = 0.75$ (friendship mechanism turned off) are compared to friendship-based contacts, class-mixing-based contacts and empirical contacts. Panel A compares the overall fraction of repeated contact links over the four base day period. Panel B instead represents the transitivity in the synthetic contacts for each base day, relative to the empirical transitivity. Panel C displays the different distributions of the local cosine similarity (distribution over all pairs of base days, and over all individuals). Finally we show in panel D the global similarities between the daily contact networks of consecutive days. Panels A, B and D are all obtained with one realisation of the synthetic contacts. Panel C is obtained with 10 realisations.

In line with the comparison of the contacts themselves, contacts with $p_{tr} = 0$ (f and p optimised) lead to distribution of outbreak sizes (Fig. J) close to those obtained with friendship-based contacts with optimised parameters. Instead, contacts with $f = 0$ (p and p_{tr} optimised) result in a distribution of outbreak size visually similar to the one obtained with class-mixing-based contacts. However, as

quantified in panels C and D, the two distributions display some differences in the median, first and third quartiles. This effect can be attributed to the increased transitivity in the transitivity preserving contact sequence compared to class-mixing-based contacts. This also leads to the larger fraction of simulations reaching at most 20% of the students for the contact sequence with $f = 0$, (p and p_{tr} optimised) compared to the rest of the contact sequences considered. This suggests that preserving transitivity while ignoring friendships may slightly hinder the transmission process.

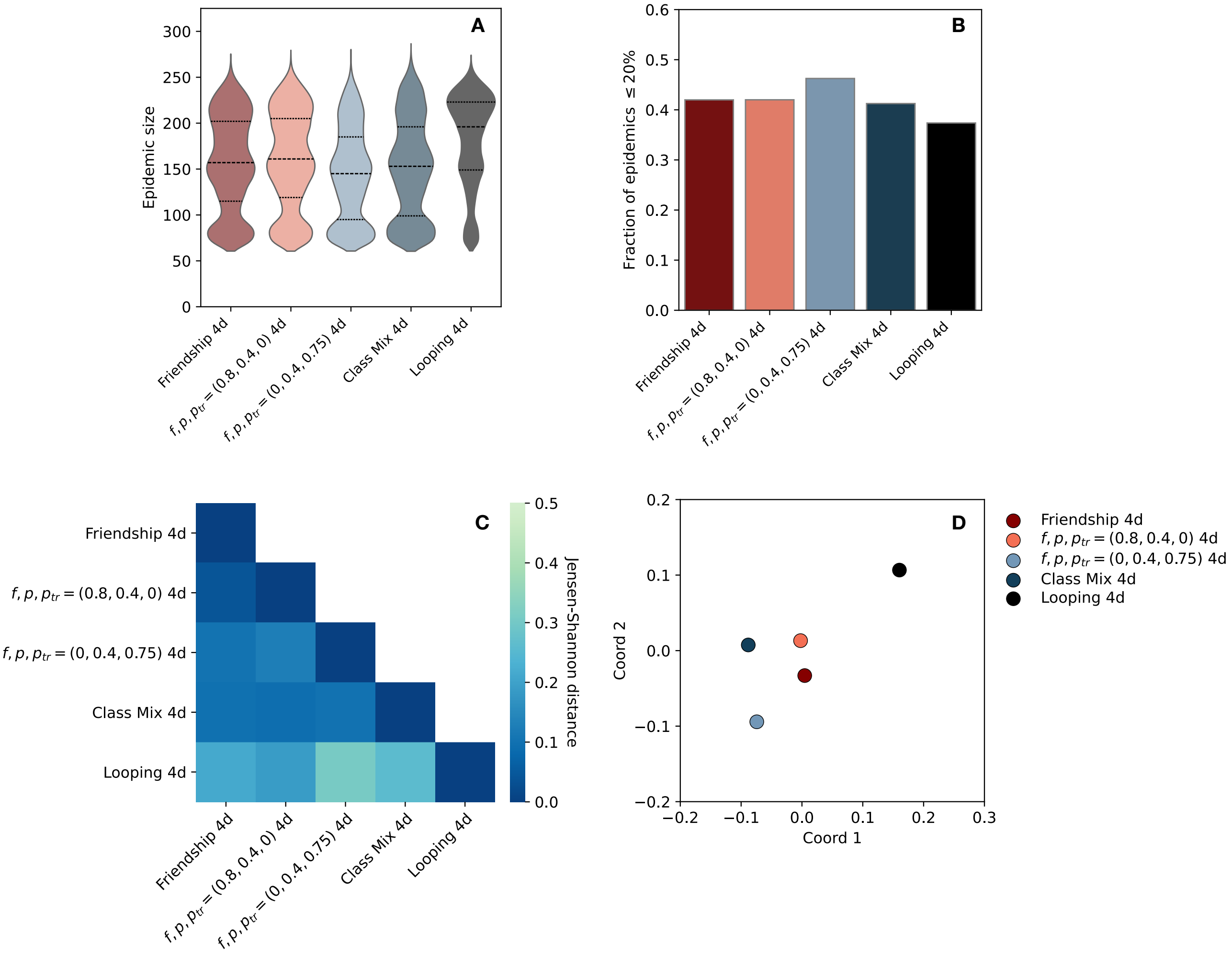

Figure J: Epidemic size distributions obtained from "Friendship 4d" contact sequences, contact sequences from the friendship-based algorithm with parameter triplets $f = 0.8$, $p = 0.4$ and $p_{tr} = 0$ (transitivity mechanism turned off), $f = 0$, $p = 0.4$ and $p_{tr} = 0.75$ (friendship mechanism turned off), "Class Mixing 4d" and "Looping 4d" contact sequences are shown in Panel A (the lines in the violin indicate the median, first and third quartiles of the distributions). Panel C instead represents the Jensen-Shannon distance for each pair of distributions from panel A. Panel D reports a multi-dimensional scaling analysis of the distance matrix from panel D. Each distribution is associated with a point in the 2d plane. Clusters of points correspond to close distributions according to the Jensen-Shannon distance, while points further apart correspond to more distant distributions. The distributions shown in panel A and compared in panels C and D are computed over simulations leading to a fraction of infected individuals larger than 20% (over 120 days) in order to better highlight differences between the distributions. Panel B shows the fraction of the number of simulations that did not reach more than 20% of the population and were therefore excluded for each contact sequence considered.

Finally, we find in panel A and B of Fig. K that infection pathways between students obtained with "Friendship 4d" and friendship contacts with $p_{tr} = 0$ show significant similarity (levels in line with the similarity between "Friendship 4d" and "Looped 4d"). The contact sequence with $f = 0$ (but p and p_{tr} optimised) leads to infection pathways between students that are closer to the "Friendship 4d" baseline

than those obtained with the “Class Mixing 4d” contacts. This shift demonstrates that preserving transitivity does impact the infection pathways between students. However, similarity levels remain low in absolute when $f = 0$, confirming the impact of the preservation of the local cosine similarity distribution. As expected, the infection pathways between classes are not affected by either aspects of the algorithm (panels C and D of Fig. K).

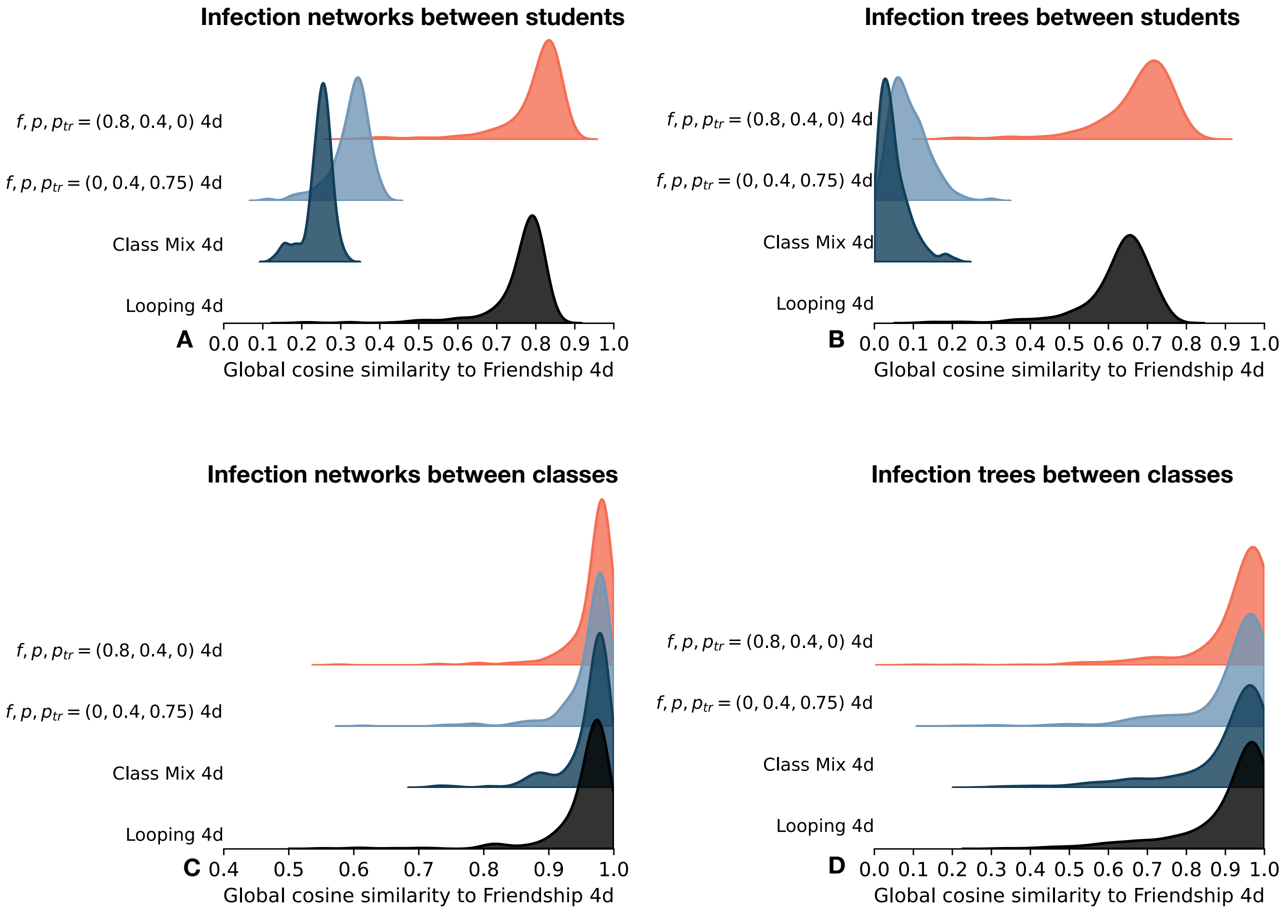

Figure K: Infection pathways obtained from the “Friendship 4d” contact sequence are compared to those obtained with contacts generated with the friendship-based algorithm with parameter triplets $f = 0.8$, $p = 0.4$ and $p_{tr} = 0$ (transitivity mechanism turned off), $f = 0$, $p = 0.4$ and $p_{tr} = 0.75$ (friendship mechanism turned off). Comparison with “Looping 4d” and “Class Mixing 4d” are reported for reference. Panel A displays the distributions (Gaussian kernel density estimations) over all seeds of the global cosine similarities $GCS(\mathcal{G}_{inf}(s, ct_a), \mathcal{G}_{inf}(s, ct_b))$ between infection networks between students. Panel B instead shows the distributions of the global cosine similarities $GCS(\mathcal{T}_{inf}(s, ct_a), \mathcal{T}_{inf}(s, ct_b))$ between infection trees instead. The bottom row instead compares infection networks (panel C) and infection trees (panel D) between classes. For each contact sequence, infection networks are obtained from 150 simulations for each seed, and each of the 325 students are successively considered as seed s.

F Additional figures

The remainder of this document is dedicated to further results complementing those presented in the core of the article.

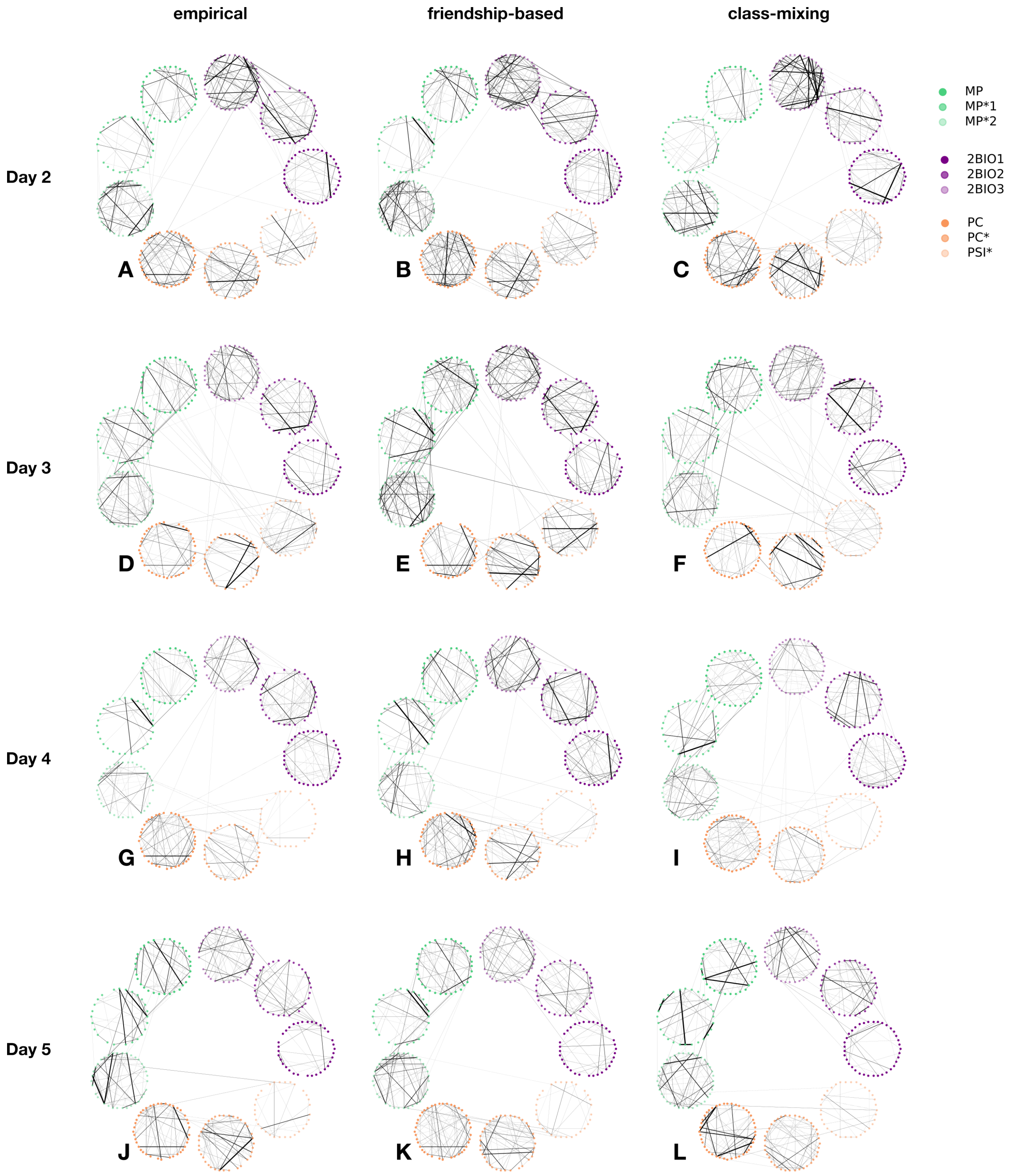

Figure L: The daily contact networks recorded on each full day of the deployment are plotted in panels A, D, G, and J. Nodes represent students, and links their contact links (omitting those cumulating less than 5 minutes). The edge thickness indicates each contact link's weight (calculated as total duration during the day). Realisations of synthetic networks based on each deployment day are plotted similarly in panels B, E, H and K (friendship-based) and in panels C, F, I, and L (class-mixing-based).

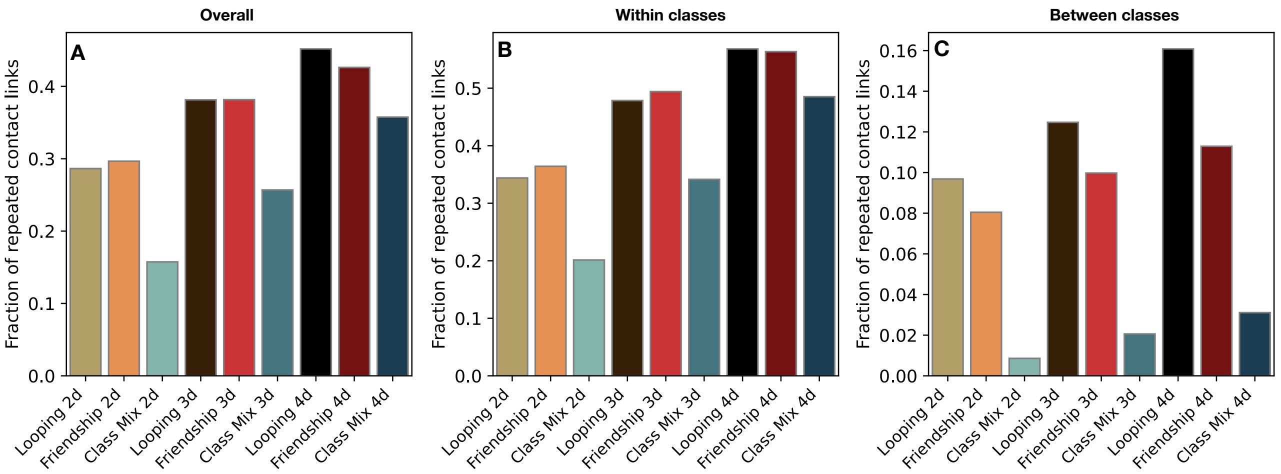

Figure M: The fraction of repeated contact links over the total number of distinct links over the base period (i.e. over two days for "2d" contact sequences, three days for "3d" sequences and four days for "4d" sequences) is shown for the different contacts considered. Panel A presents the overall fraction of repeated contact links. The fractions restricted to contact links within each class and between different classes are shown in panels B and C respectively. Results are obtained with one realisation of each day synthetically copied.

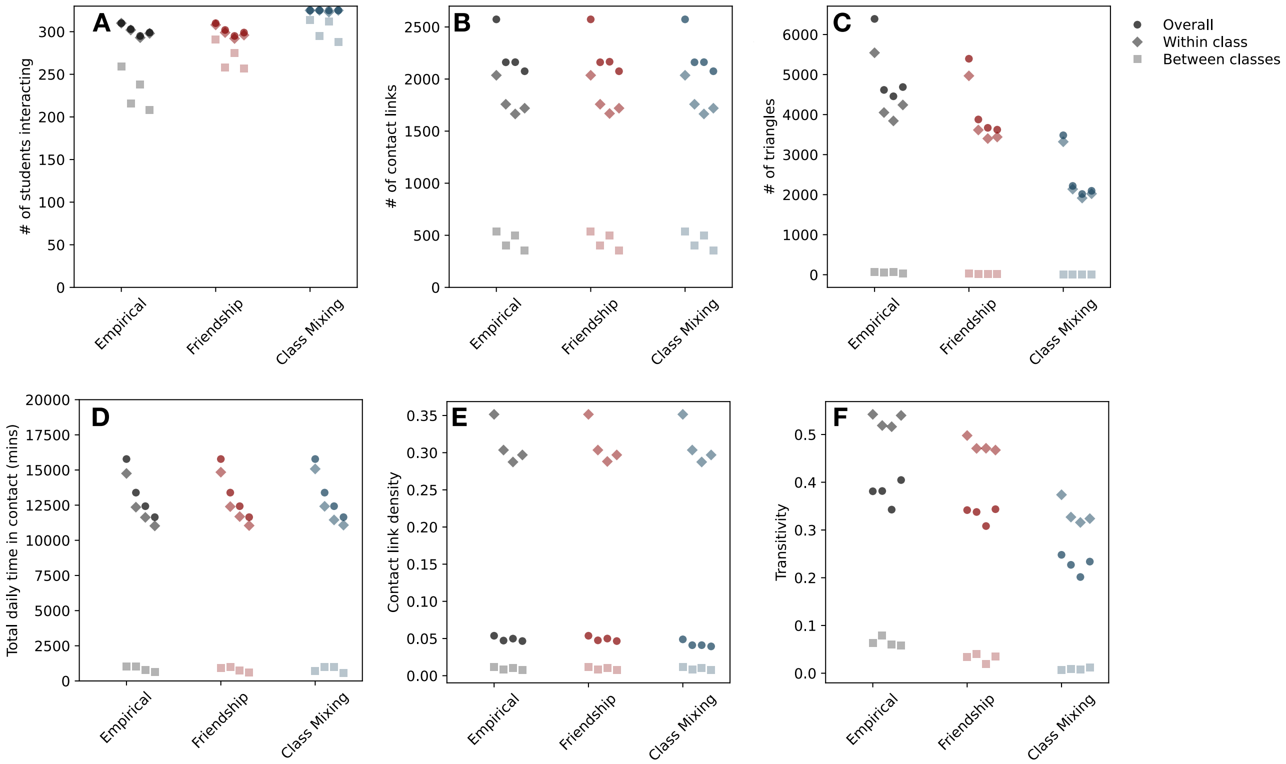

Figure N: The number of nodes, contact links and triangles are plotted respectively in panels A, B, and C for the different types of contacts. The total time spent interacting, contact link densities (ratio of the number of links observed over the number of possible contact links) and the transitivity (ratio of the number of realised triangles observed over the number of V shaped motifs and realised triangles) [2] are shown in panels D, E and F respectively. Each data points correspond to a day of the deployment (respectively an iteration based on a day of the deployment for the synthetic contacts). Each quantity is computed over all contacts, and restricted to contacts within classes and between classes.

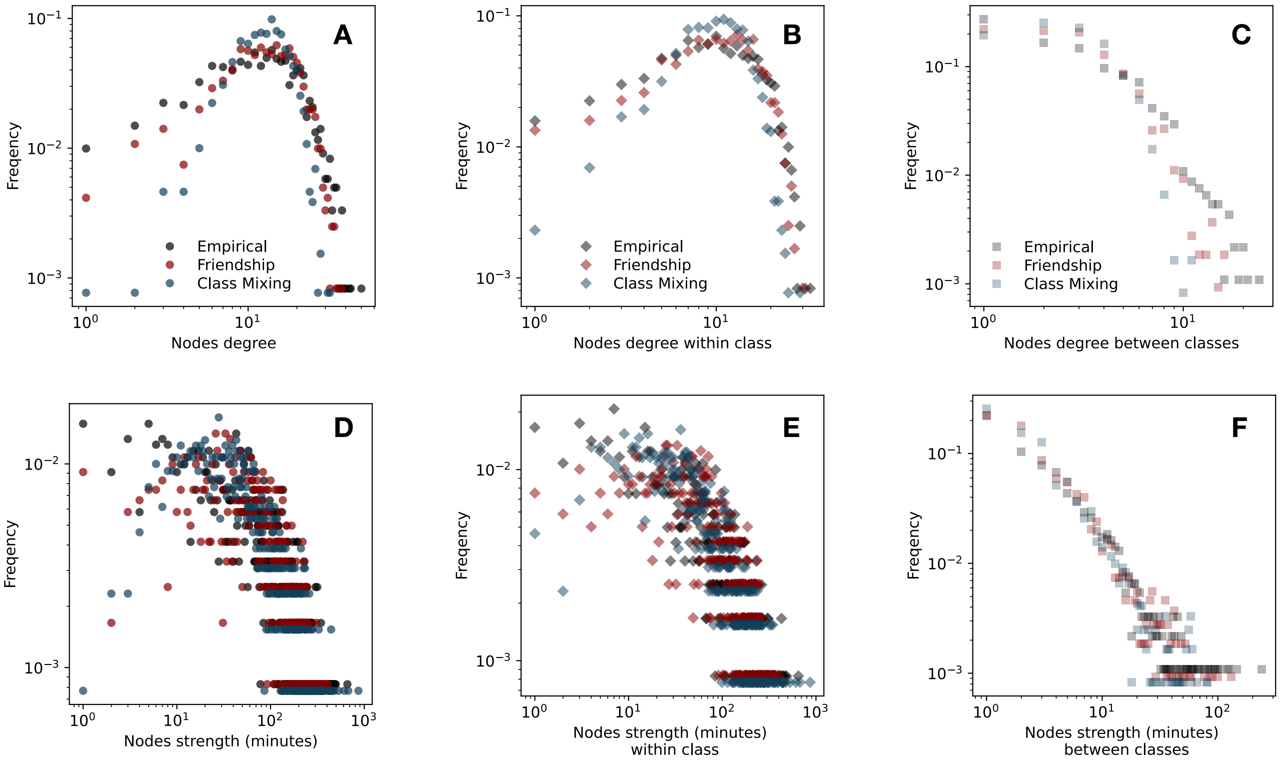

Figure O: Log-log plots of the distributions of the node degrees (panels A - C) and the node strengths (panels D - F). Each distribution is shown for all contact links (left columns), as well as for links restricted to within class contacts (centre column) and between classes contacts (right column). The degree of a node corresponds to the number of individuals it is in contact with on a given day. The strength of a node instead corresponds to the total time it spends in contact with other individuals. Distributions are computed over each day of the deployment, and each student.

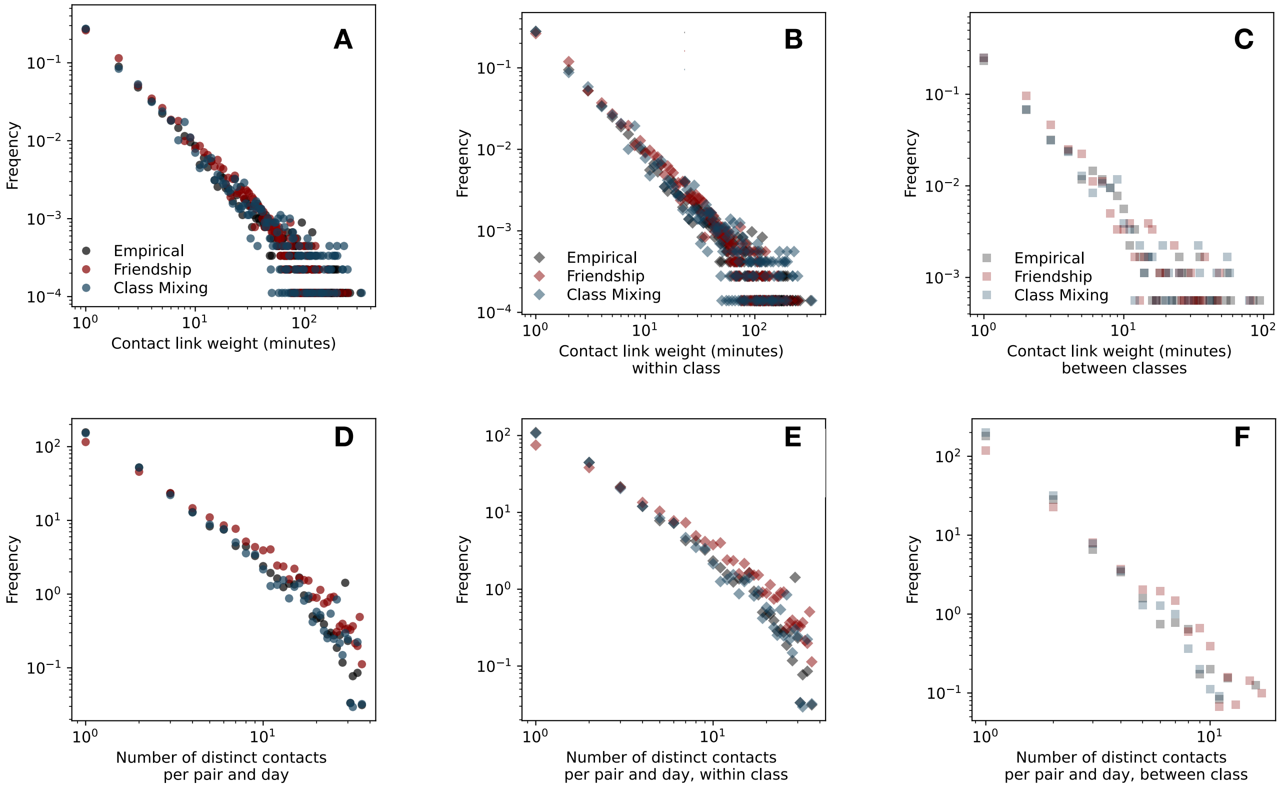

Figure P: Log-log plots of the distributions of the contact link weights are shown in panels A - C. Panels D - F instead show log-log plots of the distributions of the number of distinct contact events per day and pair of students in contact. This corresponds to the number of times an infection can occur for each day and each pair. Each distribution is shown for all contact links (left columns), as well as for links restricted to within class contacts (centre column) and between classes contacts (right column). Contact link weights are given by the daily cumulated contact duration for each pair. Distributions are computed over each day of the deployment, and each link.

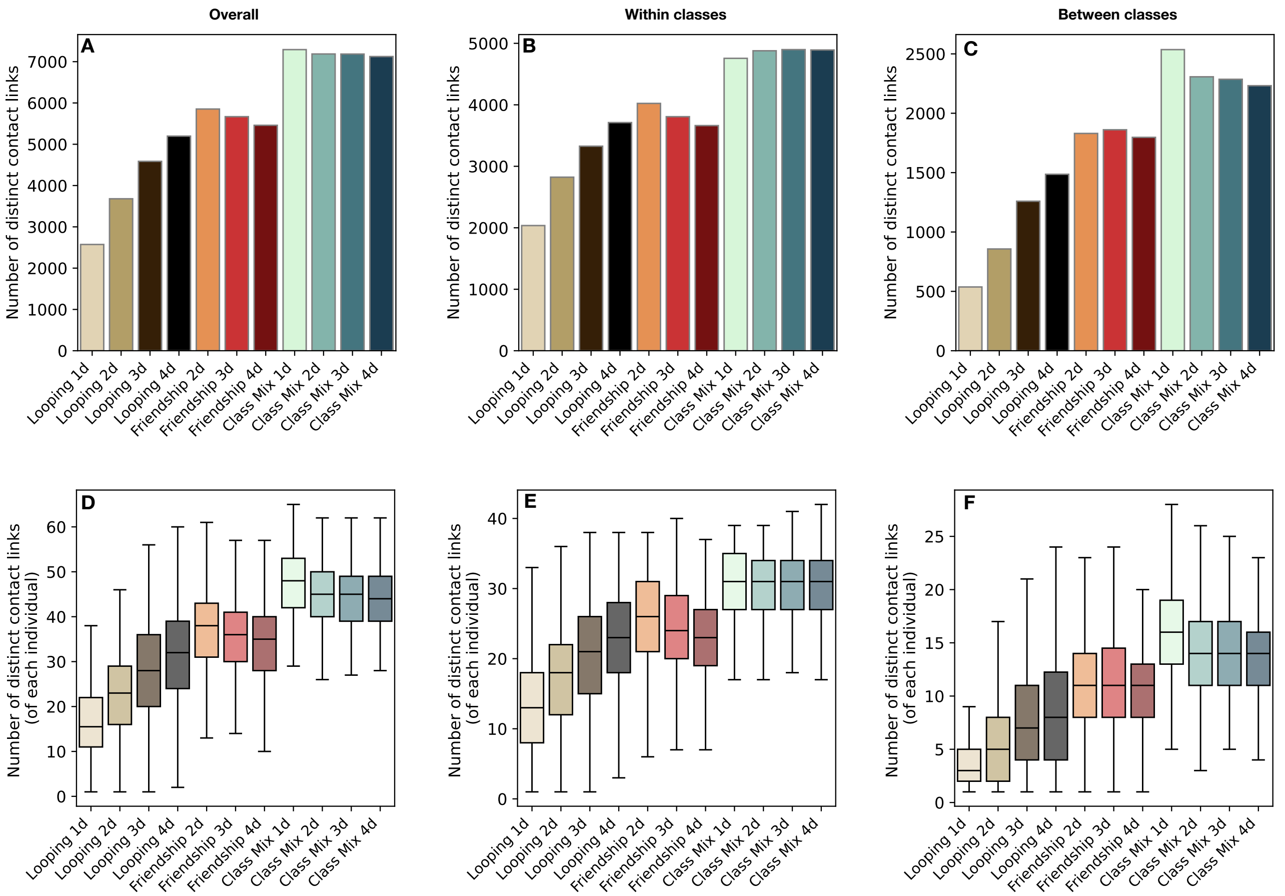

Figure Q: The total numbers of distinct pairs of individuals in contact over one week (five school days) in each contact sequence is plotted in panel A. Panels B and C instead display the equivalent numbers restricted to contacts within classes and between classes (respectively). The distribution of the weekly number of distinct contact links of each individual is instead displayed as a boxplot in panel D for each contact sequence. Panels E and F again display the equivalent distribution for the within classes and between classes contacts.

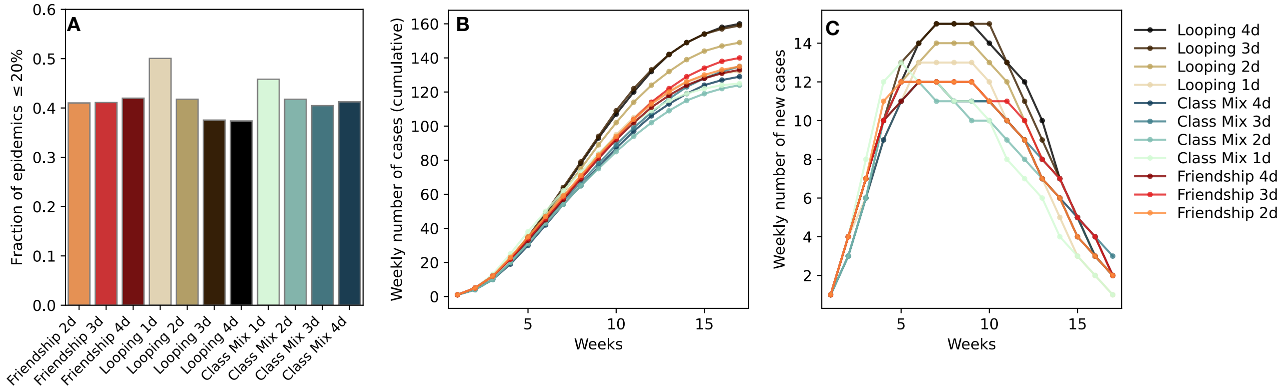

Figure R: We show in panel A the fraction of simulations for each contact sequence, all seeds combined, that lead to less than 20% of infected individuals by the end of the 120 days considered. Panels B and C display the cumulative weekly number of cases (median) and the weekly number of cases (median), obtained from outbreaks that lead to more than 20% of infected students.

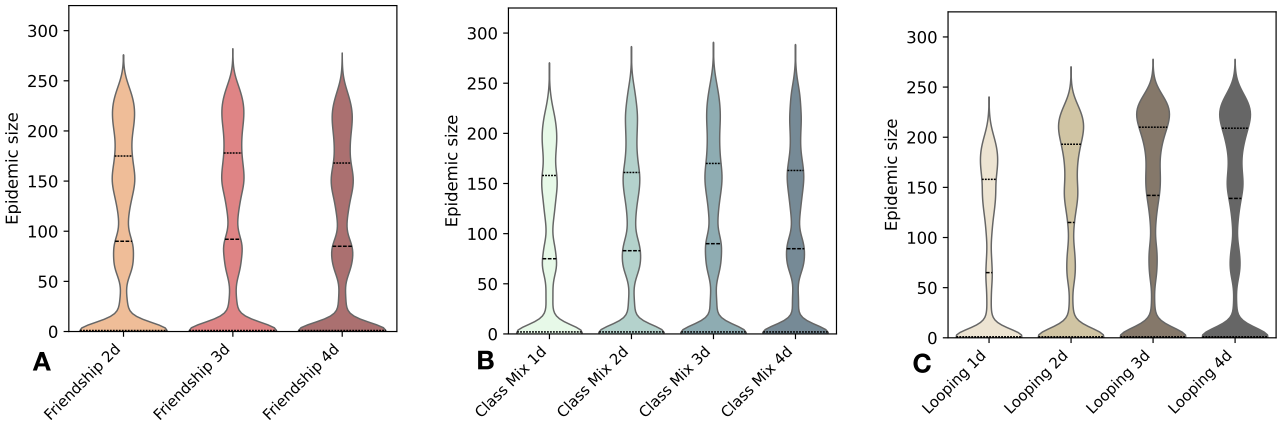

Figure S: Comparison of the epidemic size distributions obtained from different contact sequences using *all* simulations regardless of the fraction of the population infected. Distributions obtained from friendship-based contacts are shown in panel A, class-mixing-based contacts in panel B and looped contacts in panel C.

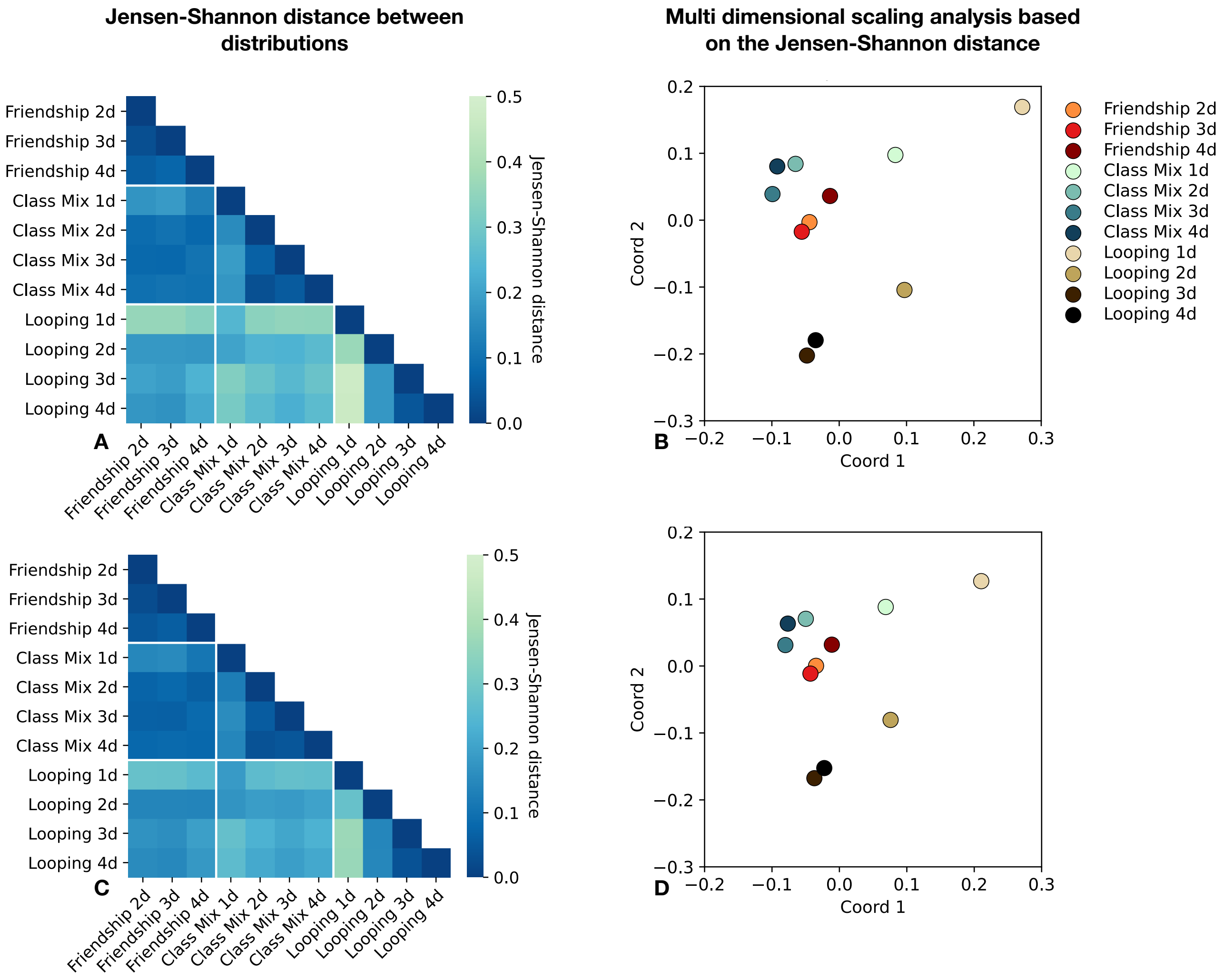

Figure T: The Jensen-Shannon distance is shown for epidemic size distributions obtained from all contact sequences in panel A and C. Panels B and D instead represents a multi-dimensional reduction of the pairwise distances into the 2 dimensional plane. Each distribution is associated a point. Clusters of points correspond to close distributions according to the Jensen-Shannon distance, while points further apart correspond to more distant distributions. Panels A and B include simulations leading to outbreaks with more than 20% of infected students at final size, while panels C and D include all simulations.

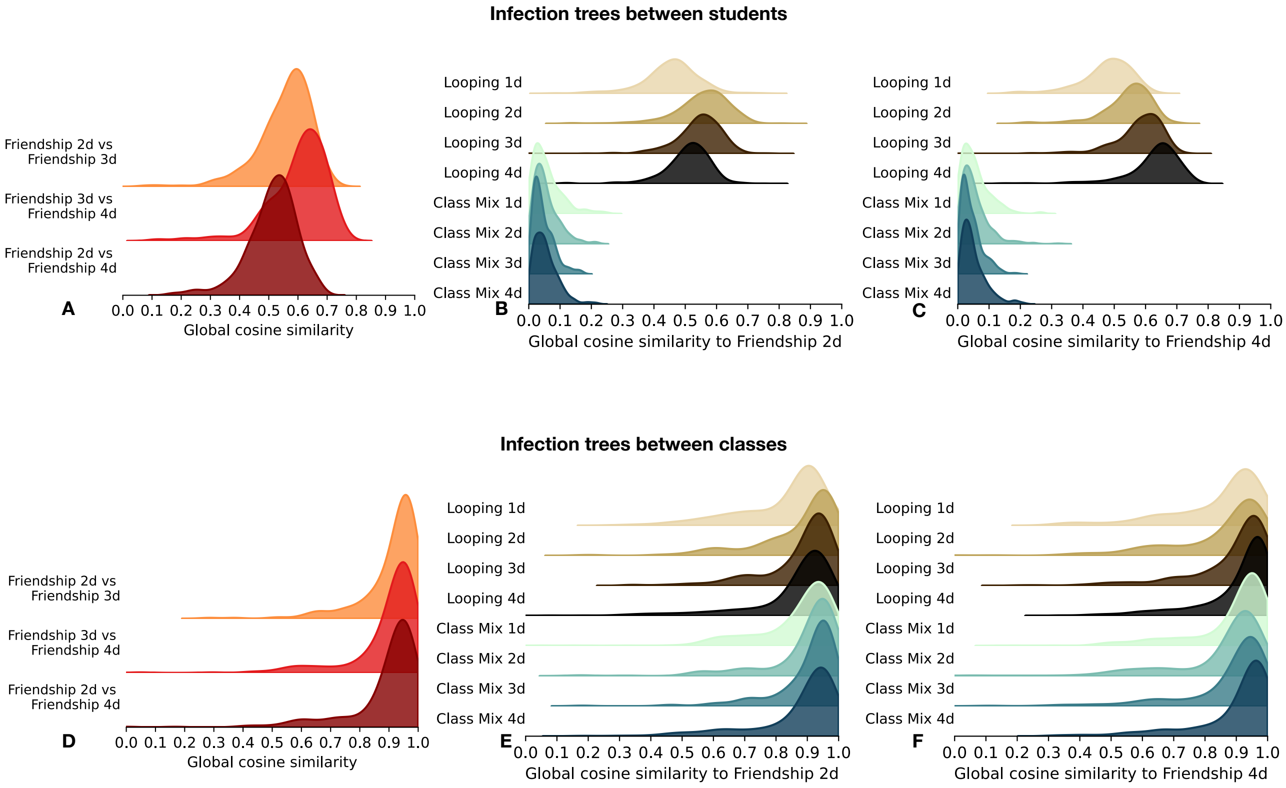

Figure U: Panels A - C compare infection trees between students while panels D - F compare infection trees with classes. For both rows: panels A and D show the respective distributions of the global cosine similarity $GCS(\mathcal{T}_{inf}(s, ct_a), \mathcal{T}_{inf}(s, ct_b))$ over all seeds indexed by s for pairs of underlying friendship-based contact sequences ct_a and ct_b ("Friendship 2d" , "Friendship 3d" and "Friendship 4d") (trees between students in panel A, trees between classes in Panel D). Panels B, C (trees between students) and panels E and F (trees between classes) instead compare trees from class-mixing-based and looped contacts to a fixed friendship-based baseline. The distribution over all seeds indexed by s of $GCS(\mathcal{T}_{inf}(s, ct_a), \mathcal{T}_{inf}(s, ct_b))$ are shown for different contact sequences ct_a among class-mixing-based and looped contacts, with ct_b fixed to "Friendship 2d". Similarly panels C and F show equivalent distributions, now with ct_b fixed to "Friendship 4d". For each contact sequence, trees are obtained from 150 simulations, with each of the 325 students considered as seed.

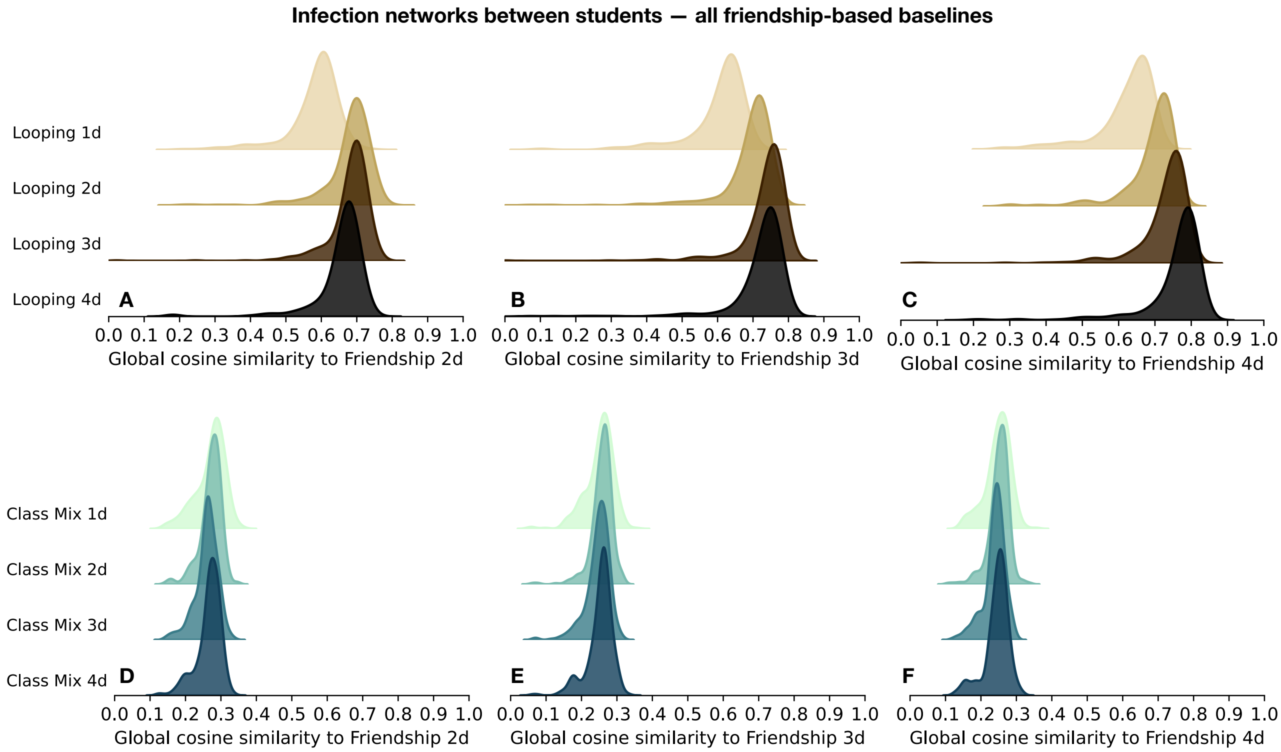

Figure V: Comparison of infection networks obtained from friendship-based contact sequences to looped contacts (panel A) and class-mixing-based contacts (panel B). For each pair ct_a, ct_b of contact sequences considered, Gaussian kernel density estimations for the distribution over all seeds s of the seed-based global cosine similarity between infection networks, $GCS(\mathcal{G}_{inf}(s, ct_a), \mathcal{G}_{inf}(s, ct_b))$ are plotted.

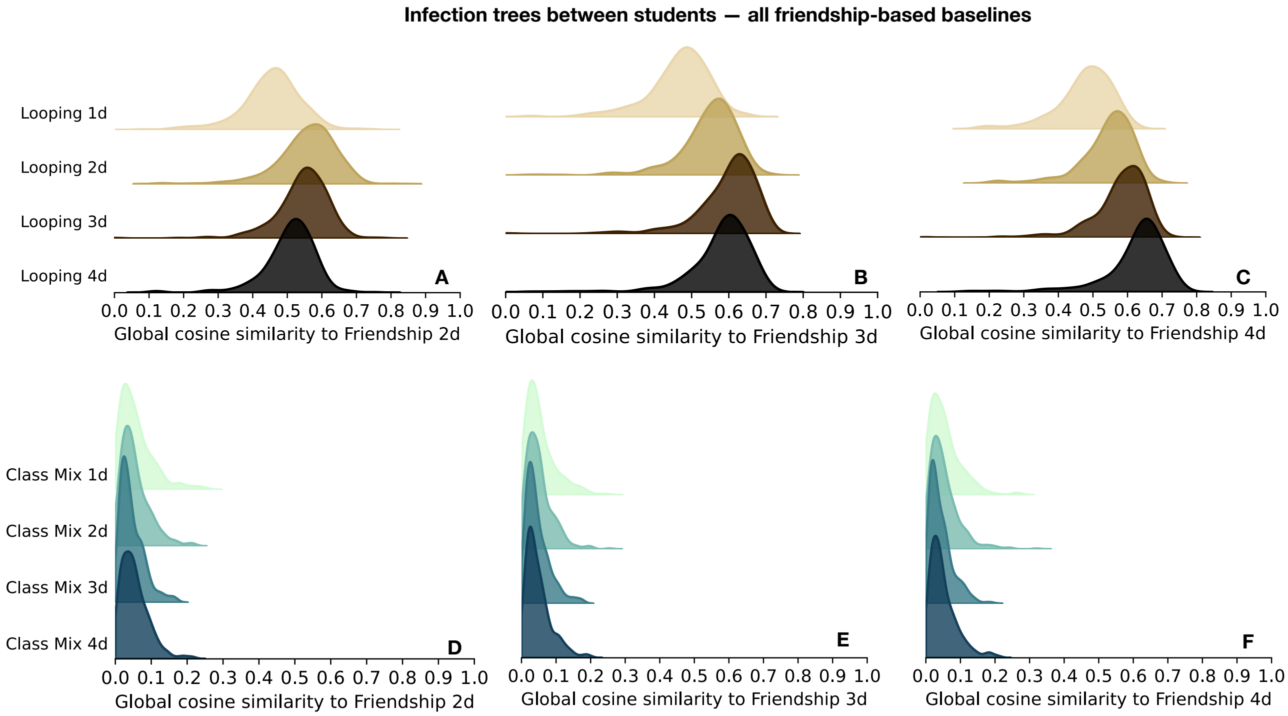

Figure W: Comparison of infection trees obtained from friendship-based contact sequences to looped contacts (panel A) and class-mixing-based contacts (panel B). For each pair ct_a, ct_b of contact sequences considered, Gaussian kernel density estimations for the distribution over all seeds s of the seed-based global cosine similarity between infection trees, $GCS(\mathcal{T}_{inf}(s, ct_a), \mathcal{T}_{inf}(s, ct_b))$, are plotted.

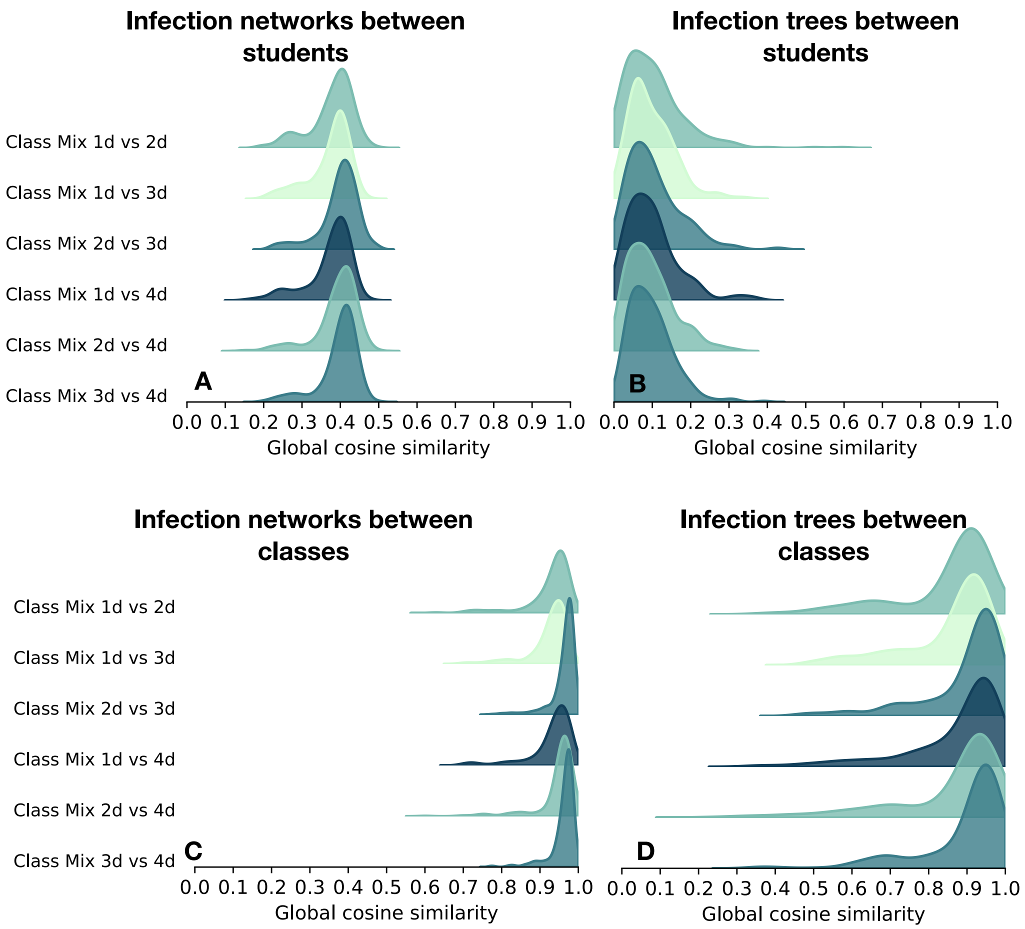

Figure X: Comparison of infection networks (panels A and C) and trees (panels B and D) obtained from class-mixing-based contact sequences built from different numbers of base days. Panels A and B compare infection pathways between students while panels C and D compare pathways between classes. For each pair ct_a, ct_b of contact sequences considered, Gaussian kernel density estimations for the distribution over all seeds s of the seed-based global cosine similarity between infection networks, $GCS(\mathcal{G}_{inf}(s, ct_a), \mathcal{G}_{inf}(s, ct_b))$, are plotted in panels A (between students) and C (between classes). Equivalent distributions are shown for infection trees in panels B (between students) and D (between classes).

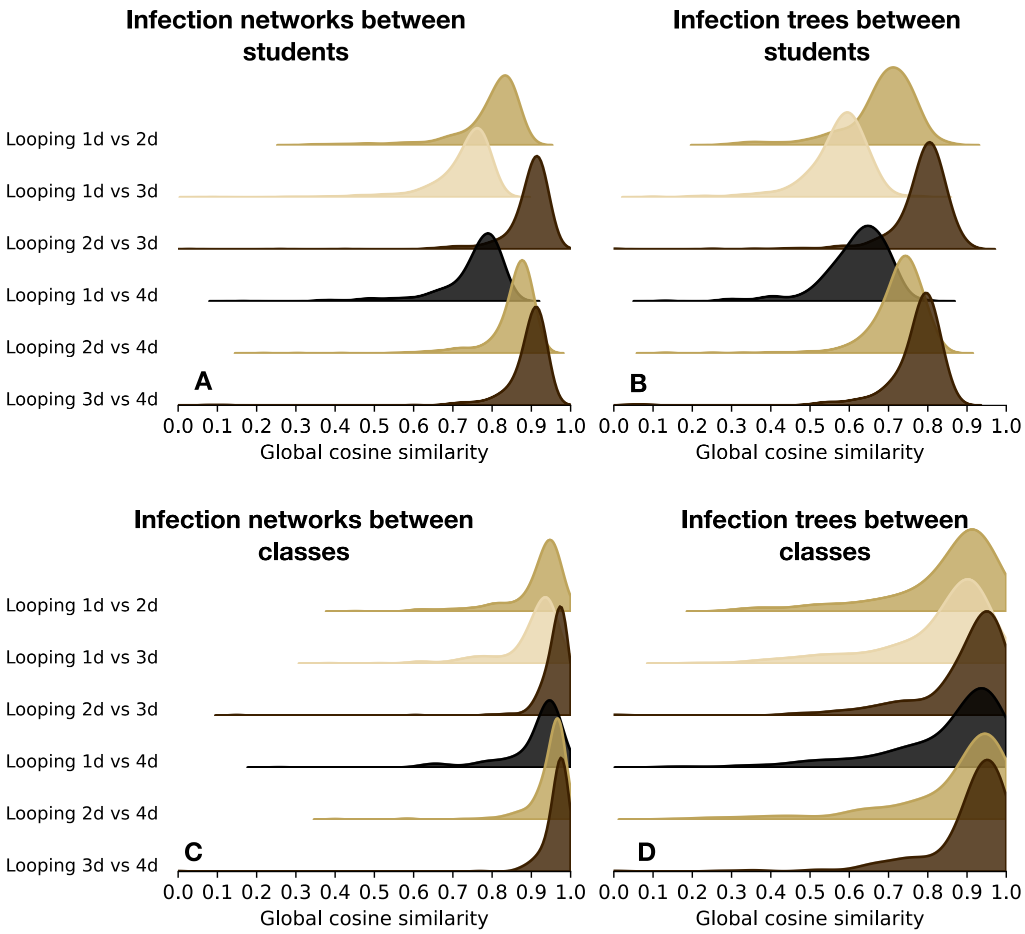

Figure Y: Comparison of infection networks (panels A and C) and trees (panels B and D) obtained from looped contact sequences built from different numbers of base days. Panels A and B compare infection pathways between students while panels C and D compare pathways between classes. For each pair ct_a, ct_b of contact sequences considered, Gaussian kernel density estimations for the distribution over all seeds s of the seed-based global cosine similarity between infection networks, $GCS(\mathcal{G}_{inf}(s, ct_a), \mathcal{G}_{inf}(s, ct_b))$, are plotted in panels A (between students) and C (between classes). Equivalent distributions are shown for infection trees in panels B (between students) and D (between classes).

References

1. Mastrandrea R, Fournet J, Barrat A. Contact patterns in a high school: a comparison between data collected using wearable sensors, contact diaries and friendship surveys. PloS one. 2015;10(9):e0136497.

2. nx.transitivity - NetworkX v3.3 Manual; [Cited 2024 October 7]. Available from: `https://networkx.org/documentation/stable/reference/algorithms/generated/networkx.al`

`gorithms.cluster.transitivity.html`.

3. scipy.spatial.distance.jensenshannon - SciPy v1.12.0 Manual; [Cited 2024 October 7]. Available from: `https://docs.scipy.org/doc/scipy/reference/generated/scipy.spatial.distance.jensenshannon.html`.

4. Lin J. Divergence measures based on the Shannon entropy. IEEE Transactions on Information theory. 1991;37(1):145–151.

5. Kullback S, Leibler RA. On information and sufficiency. The annals of mathematical statistics. 1951;22(1):79–86.

6. Endres DM, Schindelin JE. A new metric for probability distributions. IEEE Transactions on Information theory. 2003;49(7):1858–1860.

7. Levin DA, Peres Y. Markov chains and mixing times. vol. 107. American Mathematical Soc.; 2017.

8. Beran R. Minimum Hellinger distance estimates for parametric models. The annals of Statistics. 1977; p. 445–463.

9. Colosi E, Bassignana G, Contreras DA, Poirier C, Boëlle PY, Cauchemez S, et al. Screening and vaccination against COVID-19 to minimise school closure: a modelling study. The Lancet Infectious Diseases. 2022;22(7):977–989.

10. Colosi E, Bassignana G, Barrat A, Lina B, Vanhems P, Bielicki J, et al. Minimising school disruption under high incidence conditions due to the Omicron variant in France, Switzerland, Italy, in January 2022. Eurosurveillance. 2023;28(5):2200192.

11. Contreras DA, Colosi E, Bassignana G, Colizza V, Barrat A. Impact of contact data resolution on the evaluation of interventions in mathematical models of infectious diseases. Journal of the Royal Society Interface. 2022;19(191):20220164.

12. Piontti APY, Gomes MFDC, Samay N, Perra N, Vespignani A. The infection tree of global epidemics. Network Science. 2014;2(1):132–137.

13. Chu YJ, Liu TH. On the shortest arborescence of a directed graph. Scientia Sinica. 1965;14:1396–1400.

14. Edmonds J, et al. Optimum branchings. Journal of Research of the national Bureau of Standards B. 1967;71(4):233–240.

15. Hagberg A, Swart PJ, Schult DA. Exploring network structure, dynamics, and function using NetworkX. Los Alamos National Laboratory (LANL), Los Alamos, NM (United States); 2008.

16. nx.minimum-spanning-arborescence - NetworkX v3.3 Manual; [Cited 2024 October 7]. Available from: https://networkx.org/documentation/stable/reference/algorithms/generated/networkx.algorithms.tree.branchings.minimum_spanning_arborescence.html.

17. Bastian M, Heymann S, Jacomy M. Gephi: an open source software for exploring and manipulating networks. In: Proceedings of the international AAAI conference on web and social media. vol. 3; 2009. p. 361–362.
