# Supplementary material for: Preserving friendships in school contacts: An algorithm to construct synthetic temporal networks for epidemic modelling
Source: PLoS Comput Biol. 2024 Dec 9;20(12):e1012661. doi: 10.1371/journal.pcbi.1012661 (PMC11676524; doi:10.1371/journal.pcbi.1012661)
Supplement: Attachment — Submitted filename: Rebuttal friendships-2.pdf [file pcbi.1012661.s002.pdf]

Preserving friendships in school contacts: an algorithm to construct synthetic temporal networks for epidemic modelling by Calmon et al.

Point-by-point response to the Reviewers

Reviewer #1

Study explores the use of an algorithm to recreate contact networks over multiple days via a mix of repeated contacts and random casual contacts. Some minor comments
We thank the Reviewer for the attention they have given our manuscript, and the constructive comments they have provided us.

Line 31, “clear impact on the infection pathways between students” this phrasing is unclear. The details are elaborated in the paper but abstract should capture specific details of key findings.

This phrase has been replaced with *“leads to different infection pathways between individual students”* in the resubmitted manuscript.

2. Figure 1 panel 2, the details of the data set was illustrated but it's hard to interpret the meaning. Why not provide a sample table of values and illustrate the connections between persons e.g. A, B, C. There can be a column to illustrate the corresponding colour and weight of the link.

We thank the Reviewer for their feedback and suggestions. We have modified Figure 1 in order to include several tables in panel 2 of the figure, to show examples of each type of data used in the algorithm. The updated figure is shown below.

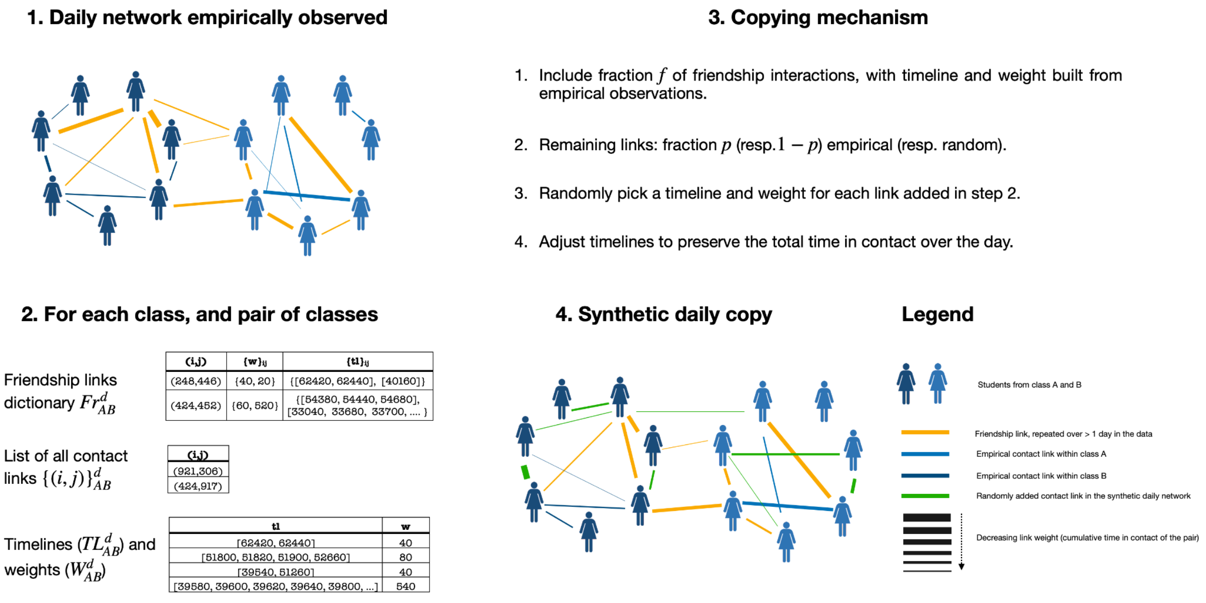

***Figure 1.** Schematic representation of the friendship-based approach to synthetic contacts generation. Daily high-resolution empirical contact networks, schematically represented in panel 1, form the starting point (input) of the method. Friendship links are highlighted in orange, while blue links correspond to empirical links occurring only on a single day. Panel 2 details the*

different lists required for the algorithm. Examples of entries are shown. Note that the timelines (lists of timestamps) are expressed in seconds from midnight on the initial day of the deployment, and the weights ("w") are expressed in seconds. These lists are specific for each class and pair of classes, and each base day. When assigning randomly a timeline and weight to contact links (step 3 of panel 3), an entry of the third table is drawn. The algorithm operates class by class, and pair of classes by pair of classes, as summarised in panel 3, to generate synthetic contact networks, schematically represented in panel 4. These networks inherit properties from the empirical contact networks, such as the number of links per class and between each pair of classes, a fraction of the friendship links (depicted in orange) and of the non-repeated links (depicted in blue). These links are complemented by random links (depicted in green) that were not necessarily observed in the base day.

3. Analysis of COVID-19 transmission and the different vaccine strategies was unnecessary given that the focus of the paper is on recreating contact networks. Instead a simple transmission model would suffice. Adding too much disease characteristics would mask the differences between the networks.

We thank the reviewer for their feedback. As the Reviewer points out, this amount of details may not be necessary to theoretically compare empirical and synthetic contacts. However, elements such as vaccination of the population or the characteristics of COVID-19 transmission are essential features of realistic transmission models (i.e. to investigate different testing strategies in schools using high resolution contacts, see Colosi et al, The Lancet Infectious Diseases 2022). Our present analysis of a COVID-19 transmission model aims to illustrate the use of the designed contacts in a context similar to the ones in which they will be most useful in the future.

In our resubmitted manuscript, we have now revised the text around line 286 in order to remove the phrasing "*The impact of vaccination was furthermore modelled…*" and focus instead on the characteristics of the population modelled and the effect of vaccination on the transmission probability in those students. The revised text reads as follows.

> *We model a vaccinated student population (homogeneous 50% coverage) by reducing the relative infectiousness of vaccinated individuals by 20% and their relative susceptibility to infection by 50%.*

4. The infection risk from multiple 20 sec interactions vs a prolong interaction might differ. Would be interesting to see if there's any variation between the networks on this. From the current plots, the measure of similarity is based on cumulative contact link weights. If the risk of infection "resets" at every exposure (i.e. risk of infection now is independent on previous interactions), then this might change the findings.

The Reviewer correctly identified that our optimization procedure aims to reproduce the distribution of local cosine similarity in the contacts, which is calculated using the cumulative contact link weights. Indeed, each distinct contact (defined within a 15 min window) corresponds in the simulations to a reset of the infection risk in the pair. The comparison of the different contact networks using the cosine similarity only takes the cumulative time in contact into account.

However, the design of the algorithm ensures that all networks are equivalent from this perspective. Indeed, we assign to each edge in the synthetic contact networks a complete

timeline (i.e. a fixed set of distinct contacts throughout the day) drawn from the empirically observed set on each day. Consequently, the contact links in all contact networks have statistically similar distributions of timelines. This applies to all contact sequences considered. Consequently, this aspect does not differ between the different networks.

We have now included an additional figure (Figure 16 of the resubmitted Supplementary Information) in order to compare the distribution of the number of distinct contacts over all individuals throughout each day. This figure is reported below.

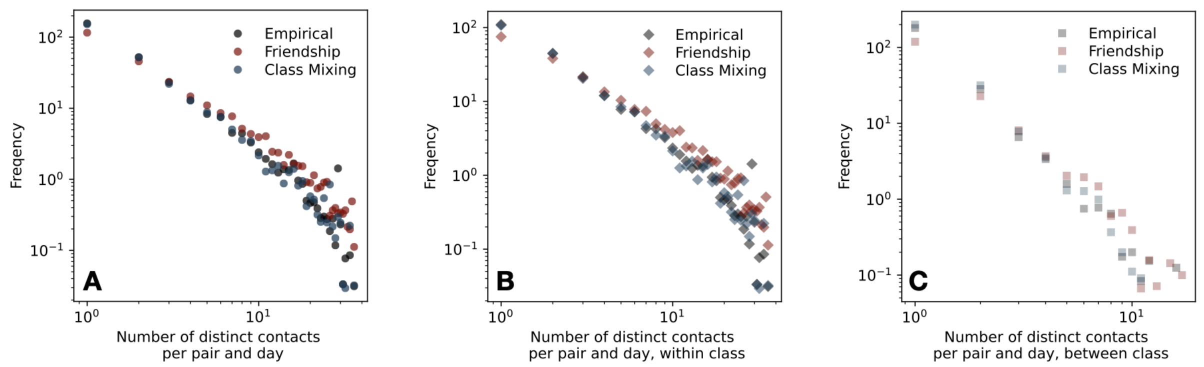

***Figure 16**: Log-log plots of the distributions of the number of distinct contact events per day and individual pair of students in contact (i.e. the number of times an infection can occur for each day and each pair). The distributions are computed over each day of the deployment, and each pair in contact for each type of network. Each distribution is shown for all contact links (panel A), as well as for links restricted to within class contacts (panel B) and between classes contacts (panel C).*

5. Line 306, why is the risk of infection not necessarily equal? Is it because of age related susceptibility or infectivity factors? Though age is not a factor mentioned in this study. Or is it due to the absence of record captured by another individual?

The Reviewer mentions the risk of infection being asymmetric. Indeed, the probability of infection from i to j is not the same as from j to i during a contact of the same duration. As the Reviewer correctly identified, all individuals considered are of similar age, and therefore this was not a factor we considered. Instead, different infection risks can occur because the two individuals can have different immunity levels (due to previous vaccination and/or natural immunity) and/or can be differently infectious (for example if they are in different stages of the disease, or have different vaccination status).

Our discussion on line 306 refers to the qualitative difference between a simulated transmission event from i to j and from j to i in simulations. It is essential to consider these two events separately when constructing transmission chains, which is why we construct directed transmission networks.

As we describe later on in the paragraph (line 309 of the original manuscript), we associate to each directed edge (i to j) of the infection network a probability of occurrence. This probability is determined both from the infection probability from i to j (determined by the model), **and** the probability of i to be infected before j, which itself depends on the position/centrality of the individual i in the contact network. For instance, if i is nearly isolated, the overall probability of i transmitting to j is very low since i is itself very unlikely to acquire the disease. This vector of probabilities allows us to compare the infection networks in Figure 5 of the manuscript.

We have now clarified the text around line 306 to improve the description of these elements. The revised text is as follows.

> *These infection networks are directed, in order to encode transmission events from i to j and from j to i separately. Both occurrences indeed correspond to different transmission chains. Each may occur with a different probability (depending on vaccination statuses of the pair, disease stages during which the contact occurs). Each directed edge l = (i,j) != (j,i) from student i to j is weighted by the occurrence probability of a transmission event from i to j (denoted p_l(s, ct_x)), estimated by the fraction of simulations in which such an event is observed (Contreras et al, PLOS Computational Biology 2024). This probability depends not only on the per unit time probability of transmission (see Supplementary Information), but also on the relative positions of i and j in the contact network. The latter impacts in particular whether i is infected before or after j.*

6. Figure 4c, when the contacts are repeated why does it result in a larger outbreak? The authors sought to explain in Line 505 but intuitively if I have 5 unchanging contacts and the probability of infection is 1, then i will always infect 5 individuals. If I have 5 changing contacts each day and the probability of infection is 0.2 over 5 days, then my average number of infections wlll also be 5 but could be as high as 25. Intuitively, repeated contacts reduces the outreach of the diseases to other parts of the network and can end prematurely due to clustering. This is a major area to elaborate further.

The Reviewer raises an important point. As the Reviewer points out, an unchanging set of contacts often reduces the outbreak size, in comparison to an equivalent number of contacts that change over time. This effect was reported in (Pung et al, MedRxiv preprint 2023; Loedy et al, Proceedings B 2024), and is strongest when contact networks are sparsest. This however does not apply in our case for the following reasons.

In the contacts in the school context considered in our study, the contact networks are sufficiently dense that this effect is insignificant once two days are looped over. In other words, the pool of susceptible individuals an infectious individual is able to transmit the infection to remains large enough in looped 2d, 3d and 4d contacts. This allows for the infection to progress unhindered even for looped “Looped 2d”, “Looped 3d”, and “Looped 4d” contacts. With “Looped 1d” contacts instead, the infection is limited by the restriction of the pool of susceptibles, resulting in smaller outbreaks.

Looping the same set of contacts induces a second opposite effect on the outbreak size. When the number of distinct contacts looped over remains diverse enough (as is the case in “looped 2d”, “looped 3d” and “looped 4d” contacts), repetition of the same set of contacts over the infectious period increases the number of unique opportunities for transmission along each contact, thus reinforcing these paths and promoting transmission.

The respective impacts of the two effects depend on the per contact probability of infection. In cases (such as the ones considered by the Reviewer) where the per contact probability is high, then the effect of the restriction of the pool of susceptible is instead dominating. In the cases considered in our study (per-unit time transmissibility of the order 10^{-2}, see Supplementary

Information), then looping contact links has instead a strong reinforcing effect along the available transmission paths.

We have now included in the Supplementary Information (see Figure 17 in the resubmitted Supplementary Information) the following additional figure showing the number of distinct contact links over one week for each contact sequence.

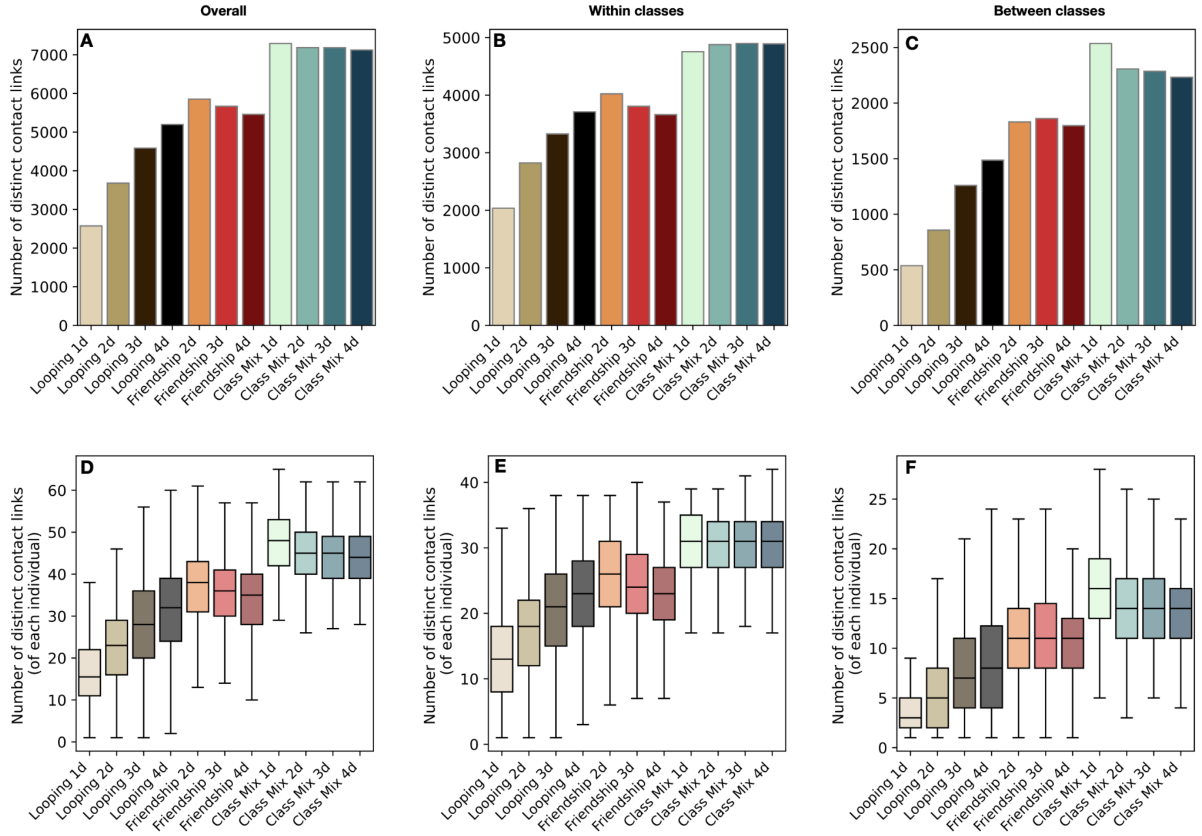

Figure 17*: The total numbers of distinct pairs of individuals in contact over one week (five school days) in each contact sequence is plotted in panel A. Panels B and C instead display the equivalent numbers restricted to contacts within classes and between classes (respectively). The distribution of the weekly number of distinct contact links of each individual is instead displayed as a boxplot in panel D for each contact sequence. Panels E and F again display the equivalent distribution for the within classes and between classes contacts.*

The paragraph of the discussion discussing these aspects has furthermore been edited for clarity.

> *Our study also highlights consequences of looping over the data, an approach commonly used to inform models with high-resolution data. We note two opposite effects at play. Firstly, looping over recorded data limits the pool of susceptible individuals an infectious individual can pass the infection to. This results in a general shift of epidemic size distributions to lower sizes. This effect is strongest when the number of distinct contacts an individual has over their infectious period is low. In the looped contacts considered, the contact networks are sufficiently dense that this effect is insignificant as soon as two days are looped over. A second effect instead causes the inflated outbreaks observed. The periodic recurrence of all contacts can reinforce transmission routes as identical contacts reoccur during the infectious period. In our simulations, we find that it dominates as soon as two days of data are looped over, yielding inflated epidemic sizes.*

Reviewer #2

Summary & Overall Impression:

In this manuscript, the authors propose two algorithms to extend short-term high-resolution contact data to longer time horizons. The main innovation in these algorithms is the possibility of preserving specific details of the contact patterns, such as class-mixing probabilities and repeated contacts, termed friendships in the manuscript. The authors demonstrate the practical relevance of preserving these features by conducting agent-based simulations of the spread of SARS-CoV-2 in a school setting based on face-to-face contact data that has been synthetically extended using their method.

The manuscript makes a significant methodological contribution to the study of temporal networks and face-to-face contact data, especially as applied to infectious disease modeling. The writing is clear and concise throughout the manuscript. The presented methods and the assessment of the influence on agent-based models are technically sound. In this context I want to highlight that the authors use a more realistic compartmental model, instead of a toy model like SIR, thereby facilitating the interpretation of their results in practically relevant contexts. I also found the comparison of outbreak sizes on the level of distributions, instead of low-dimensional summary statistics very insightful.

While this manuscript strikes me overall as a strong contribution that will be of interest to a variety of readers, I want to draw the authors' attention to the following points.

We thank the Reviewer for the time they have allocated our manuscript, and their positive feedback on our implemented methods and analysis. The Reviewer raises two especially important points, which we have now investigated. In the resubmitted manuscript, we are pleased to include substantial new results and insight in order to address these points. We hope that the Reviewer finds the manuscript improved.

Major Comments:

1. The method that the authors use to tune the algorithm's parameters is based on optimizing the Jensen-Shannon divergence between the distributions of local cosine similarities (eq. 1) of the empirical and synthetic networks. This appears to be an ad hoc choice, and I wonder whether this choice of objective function can be justified theoretically, e.g., in terms of beneficial properties for the optimization process or from the perspective of an underlying implicit probabilistic model. Providing the reader with the underlying reasoning would help them to understand the choice of parameters in applications and also facilitate follow-up research on the proposed algorithm.

We thank the Reviewer for their constructive comment. In the resubmitted Supplementary Information, we now reproduced the grid exploration and optimisation using three additional distances for sensitivity. We have also included additional details in the Supplementary Information to define the Jensen-Shannon distance, and provide some intuition regarding what it represents.

The newly conducted sensitivity analysis reads as follows.

We considered the following three additional distances to measure the similarity between LCS_{pdf,emp} and LCS_{pdf,fr}(f,p,p_tr).

The first alternative measure we considered is the total variation distance. This distance can be defined as (Levin et al, American Mathematical Soc. 2017)

$$d_{TVD}(P, Q) \;=\; \frac{1}{2}\sum_{x}|P(x) \;-\; Q(x)|$$

The value of the total variation distance for different triplets of parameters is reported in Figure 3. This distance is minimal for f=0.8, p=0.4, p_tr = 0.75 as was the case with the Jensen-Shannon distance.

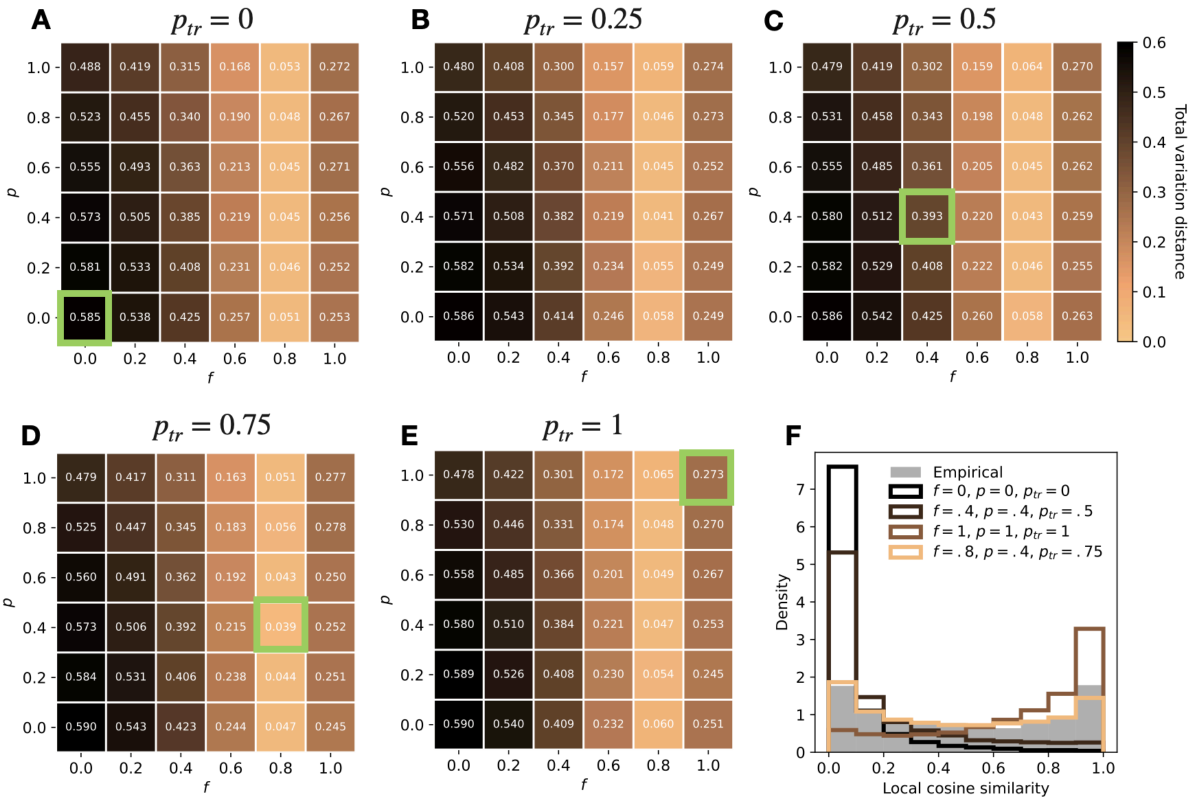

***Figure 3**: Values of the total variation distance between distributions of local cosine similarities obtained from empirical contacts (LCS_{pdf,emp}) and contacts obtained from the friendship-based approach (LCS_{pdf,fr}(f,p,p_tr)) generated with varying values of f, p (x and y axis) and p_tr (p_tr = 0, 0.25, 0.5, 0.75 and 1 in panels A, B, C, D and E) are shown in panels A to E. Panel F instead shows the distributions LCS_{pdf,emp} and LCS_{pdf,fr}(f,p,p_tr) obtained with four different triplets of parameters, highlighted in green in corresponding panels. Values shown in panels A to E are rounded to three decimals.*

The Hellinger distance is an alternative measure of similarity between two probability distributions, defined as (Beran, The annals of Statistics 1977)

$$d_{H}(P, Q) \;=\; \sqrt{2[1 \;-\; \sum_{x}\sqrt{P(x)Q(x)}]}$$

Its value for different triplets of parameters is reported in Figure 4. This distance again is minimal for f=0.8, p=0.4, p_tr = 0.75.

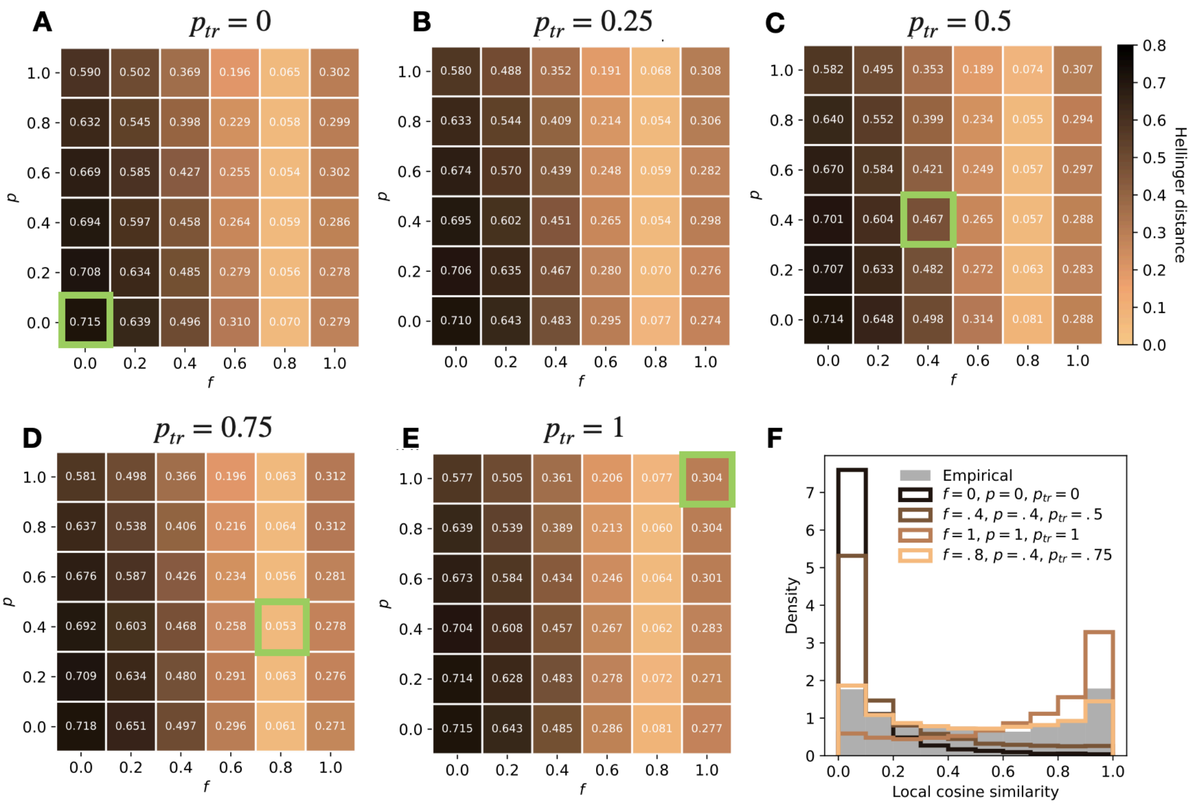

Figure 4: *Values of the Hellinger distance between distributions of local cosine similarities obtained from empirical contacts (LCS_{pdf,emp}) and contacts obtained from the friendship-based approach (LCS_{pdf,fr}(f,p,p_tr)) generated with varying values of f, p (x and y axis) and p_tr (p_tr = 0, 0.25, 0.5, 0.75 and 1 in panels A, B, C, D and E) are shown in panels A to E. Panel F instead shows the distributions LCS_{pdf,emp} and LCS_{pdf,fr}(f,p,p_tr) obtained with four different triplets of parameters, highlighted in green in corresponding panels. Values shown in panels A to E are rounded to three decimals.*

Finally, we additionally considered the Euclidean distance, defined as

$$d\ (P, Q) = \sqrt{\sum_{x}(P(x) - Q(x))^2}$$

The Euclidean distance is shown for different triplets of parameters in Figure 5. This distance is not minimal for f=0.8, p=0.4, p_tr = 0.75. Instead, its minimum corresponds to the parameter triplets f=0.8, p=0.8, p_tr = 0.25. However, the value of the Euclidean distance for the triplets optimising the remaining of the distances is very close to the minimum (0.387930 versus 0.353763 at the minimum).

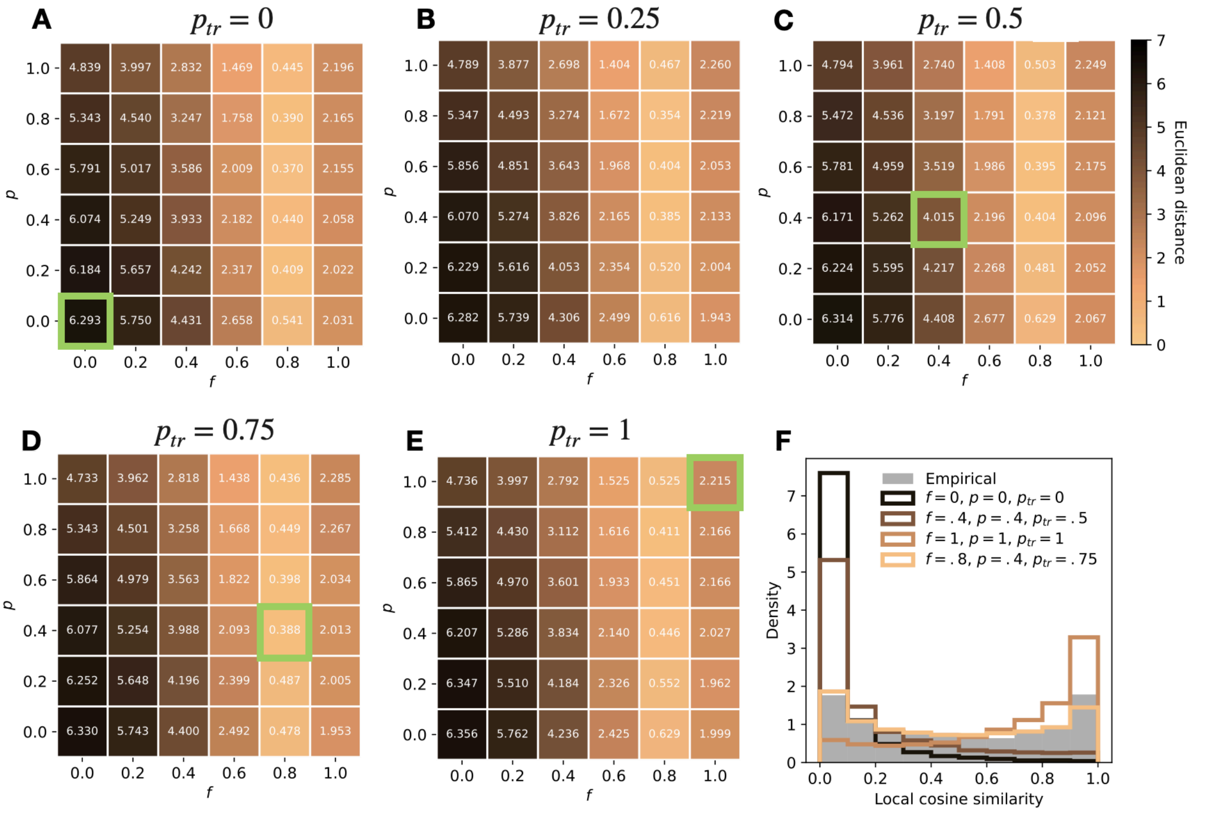

***Figure 5**: Values of the Euclidean distance between distributions of local cosine similarities obtained from empirical contacts (LCS_{pdf,emp}) and contacts obtained from the friendship-based approach (LCS_{pdf,fr}(f,p,p_tr)) generated with varying values of f, p (x and y axis) and p_tr (p_tr = 0, 0.25, 0.5, 0.75 and 1 in panels A, B, C, D and E) are shown in panels A to E. Panel F instead shows the distributions LCS_{pdf,emp} and LCS_{pdf,fr}(f,p,p_tr) obtained with four different triplets of parameters, highlighted in green in corresponding panels. Values shown in panels A to E are rounded to three decimals.*

The revised text describing the Jensen-Shannon distance now reads as follows.

> *The Jensen-Shannon distance is defined as the square root of the Jensen-Shannon divergence (Lin, IEEE Transactions on Information theory 1991), itself given by a symmetrisation of the Kullback-Leibler divergence (Kullback et al, The annals of mathematical statistics 1951). The Kullback-Leibler divergence is defined for two probability distributions P and Q as*
>
> $$D_{KL}(P,Q) = \sum_x P(x) ln(P(x)/Q(x))$$
>
> *The Kullback-Leibler divergence measures the difference from the distribution P(x) to the baseline Q(x). It can be interpreted as the relative entropy of P(x) with respect to Q(x). This measure is however asymmetric, and unbounded.*
>
> *The Jensen-Shannon divergence (Lin, IEEE Transactions on Information theory 1991) is instead given by*
> $D_{JS}(P,Q) = \frac{1}{2}(D_{KL}(P,M) + D_{KL}(Q,M))$ *where* $M(x) = \frac{1}{2}(P(x) + Q(x))$ *acts as a single baseline for both distributions. This divergence measure has the advantage of*

being symmetric, and bounded. The Jensen-Shannon distance, given by $d_{JS}(P,Q) = \sqrt{D_{JS}(P,Q)}$ is formally a metric (Endres et al, IEEE Transactions on Information theory 2003). We therefore use $d_{JS}(P,Q)$ in our analysis to measure the distance between LCS_{pdf,emp} and LCS_{pdf,fr}(f,p,p_tr) for each triplet of parameters.

2. Compared to the class-mixing-based approach the friendship-based approach makes two main modifications: It aims to maintain friendship ties and it aims to maintain network transitivity. Both network transitivity and the presence of temporal correlations between links influence how a contagion process unfolds on a temporal network. I believe exploring these effects not only in combination but also in isolation, would strengthen the manuscript by clarifying further which aspects of the empirical contact data need to be preserved and which should be randomized to obtain realistic synthetic data. Extending the analysis presented in the manuscript to the cases p_tr=0, f>0 and p_tr>0, f=0 could, for example, be a first step in this direction.

We thank the Reviewer for their comment and suggestion. We have reproduced our analysis with two additional contact sequences, obtained from the friendship-based algorithm applied with respectively f=0, p =0.4, p_tr = 0.75 (i.e. optimised parameters but no inclusion of friendship), and with f=0.8, p =0.4, p_tr = 0 (i.e. optimised parameters but no transitivity preserving mechanism). The following text and figures have been included in the resubmitted Supplementary Information.

In order to tease the individual impacts of the parameters f and p_tr, we now compare the sequences generated with the friendship-based and class-mixing-based algorithms with two new sequences obtained from the friendship-based algorithm with either f=0 (switching off the friendship mechanism) or p_tr=0 (switching off the transitivity mechanism). In both cases, the remaining two parameters are kept to their optimised values. The fitting procedure described in section 3.3 was repeated with both contact sequences in order to obtain the values of beta used for simulating the transmission model. Fitted values of beta used were respectively beta = 0.0162 for the contact sequence with f=0, p=0.4, p_tr = 0.75, and beta = 0.0196 for the contact sequence with f=0.8, p=0.4, p_tr = 0.

Comparing the contact networks themselves (Figure 9, panel A), we find that the fraction of repeated contact links over the four base days considered is affected by the friendship mechanism (f>0) but not by the transitivity preserving mechanism. Indeed, ``Friendship 4d'' and friendship-based contacts with p_tr = 0 both have very similar fractions of repeated edges. Instead, setting f=0 leads to a fraction of repeated links in line with that of the class-mixing-based contacts.

As shown in panel B, the transitivity itself is not strongly impacted by the transitivity mechanism. All contacts obtained with the friendship-based mechanism (regardless of the values of p_tr) lead to similar transitivity levels, slightly below empirical ones. This is expected. Indeed, the empirical contact networks we consider are dense, and as a result, when f=0.8 and p = 0.4 (optimised values), we already add a high number of triangles. Instead, setting f=0 and retaining p_tr=0.75, the transitivity is only ever so slightly higher

than when f=0.8, p_tr = 0. However, when neither mechanisms are in place (class-mixing-based contacts), the transitivity levels decrease significantly.

The contact sequence obtained with f=0 (but p, p_tr >0) has local cosine similarity distribution (panel C) and global day to day cosine similarity (panel D) very close to the ones of the class mixing-based contacts. This is again to be expected, as the parameter f plays a crucial role in reproducing the similarity in the contacts. Both local cosine similarity distributions and day-to-day global cosine similarities are instead not affected by the transitivity preserving mechanism.

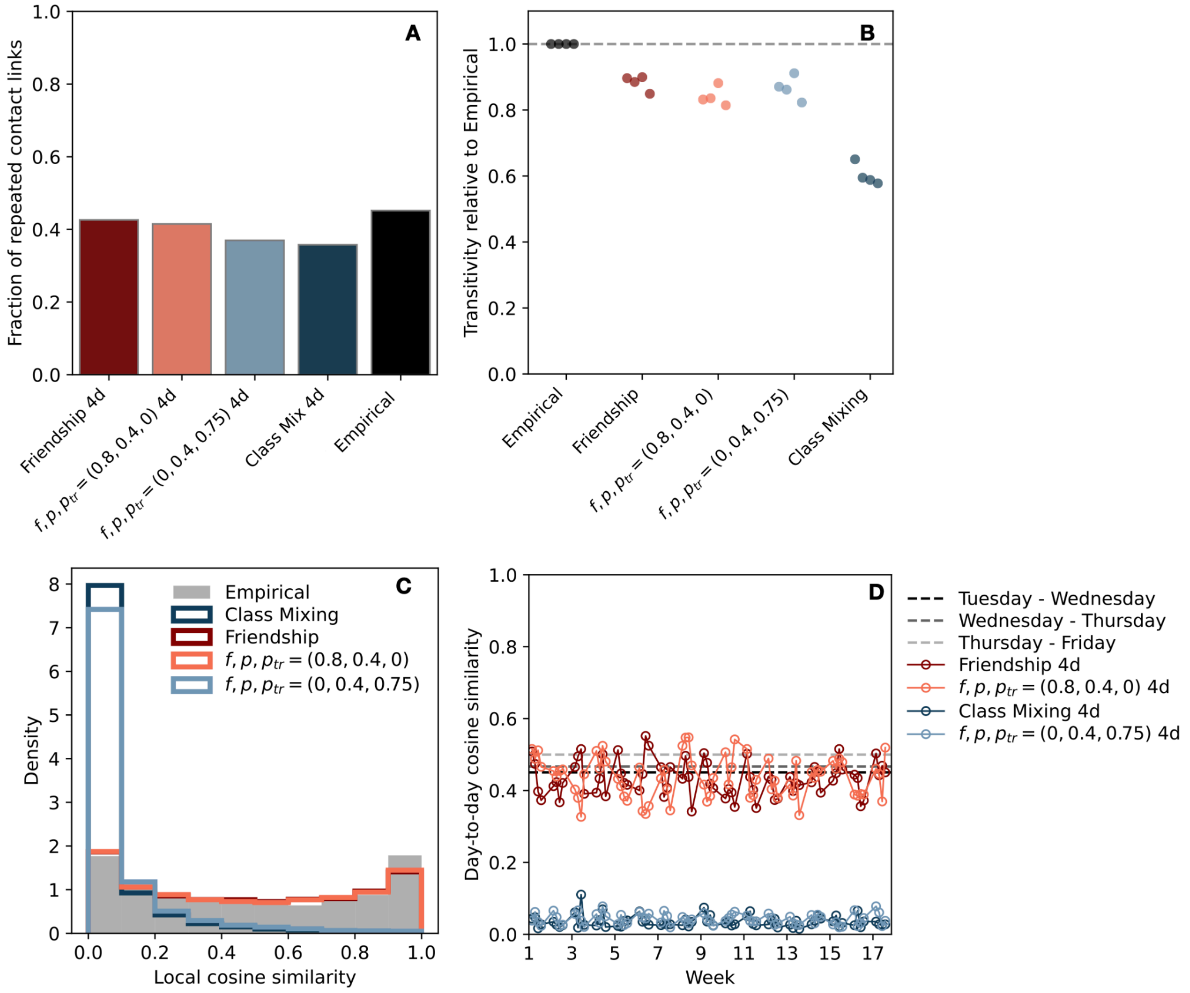

Figure 9: *Contacts from the friendship-based algorithm with parameter triplets f= 0.8, p =0.4 and p_tr = 0 (transitivity mechanism turned off), f= 0, p =0.4 and p_tr = 0.75 (friendship mechanism turned off) are compared to friendship-based contacts, class-mixing-based contacts and empirical contacts. Panel A compared the overall fraction of repeated contact links over the four base day period. Panel B instead represents the transitivity in the synthetic contacts for each base day, relative to the empirical transitivity. Panel C displays the different distributions of the local cosine similarity (distribution over all pairs of base days, and over all individuals). Finally we show in panel D the global similarities between the daily contact networks of consecutive days. Panels A, B and D are all obtained with one realisation of the synthetic contacts. Panel C is obtained with 10 realisations.*

In line with the comparison of the contacts themselves, contacts with p_tr = 0 (f and p optimised) lead to distribution of outbreak sizes (Figure 10) close to those obtained with friendship-based contacts with optimised parameters. Instead, contacts with f = 0 (p and p_tr optimised) result in a distribution of outbreak size visually similar to the one obtained with class-mixing-based contacts. However, as quantified in panels C and D, the two distributions display some differences in the median, first and third quartiles. This effect can be attributed to the increased transitivity in the transitivity preserving contact sequence compared to class-mixing-based contacts. This also leads to the larger fraction of simulations reaching at most 20% of the students for the contact sequence with f=0, (p and p_tr optimised) compared to the rest of the contact sequences considered. This suggests that preserving transitivity while ignoring friendships may slightly hinder the transmission process.

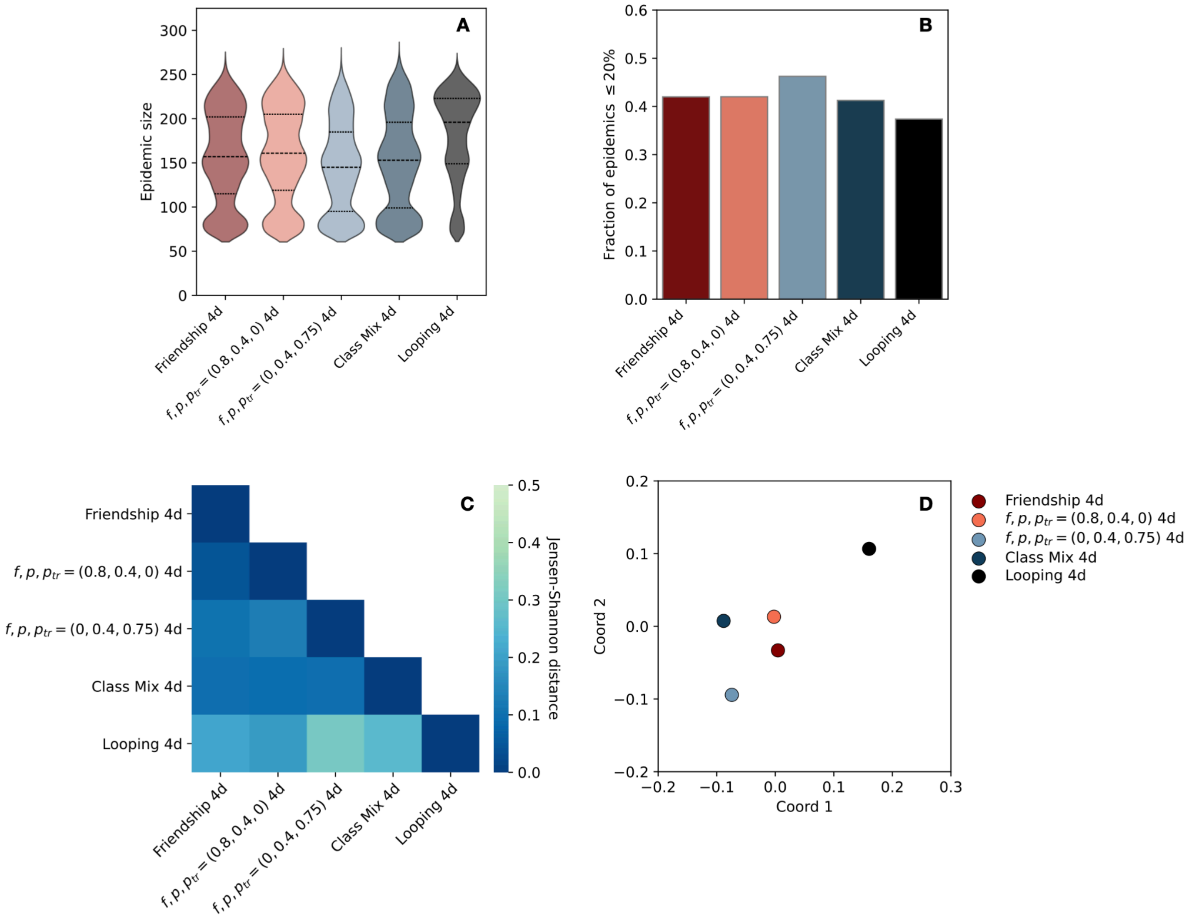

Figure 10: *Epidemic size distributions obtained from ``Friendship 4d” contact sequences, contact sequences from the friendship-based algorithm with parameter triplets f= 0.8, p =0.4 and p_tr = 0 (transitivity mechanism turned off), f= 0, p =0.4 and p_tr = 0.75 (friendship mechanism turned off), ``Class Mixing 4d” and ``Looping 4d” contact sequences are shown in Panel A (the lines in the violin indicate the median, first and third quartiles of the distributions). Panel C instead represents the Jensen-Shannon distance for each pair of distributions from panel A. Panel D reports a multi-dimensional scaling analysis of the distance matrix from panel D. Each distribution is associated with a point in the 2d plane. Clusters of points correspond to close distributions according to the Jensen-Shannon distance, while points further apart correspond to more distant distributions. The distributions shown in panel A and compared in panels C and D*

are computed over simulations leading to a fraction of infected individuals larger than 20% (over 120 days) in order to better highlight differences between the distributions. Panel B shows the fraction of the number of simulations that did not reach more than 20% of the population and were therefore excluded for each contact sequence considered.

Finally, we find in panel A and B of Figure 11 that infection pathways between students obtained with ``Friendship 4d" and friendship contacts with p_tr = 0 show significant similarity (levels in line with the similarity between ``Friendship 4d" and ``Looped 4d"). The contact sequence with f=0 (but p,p_tr optimised) leads to infection pathways between students that are closer to the ``Friendship 4d" baseline than those obtained with the ``Class Mixing 4d" contacts. This shift demonstrates that preserving transitivity does impact the infection pathways between students. However, similarity levels remain low in absolute when f=0, confirming the impact of the preservation of the local cosine similarity distribution. As expected, the infection pathways between classes are not affected by either aspects of the algorithm (panels C and D Figure 11).

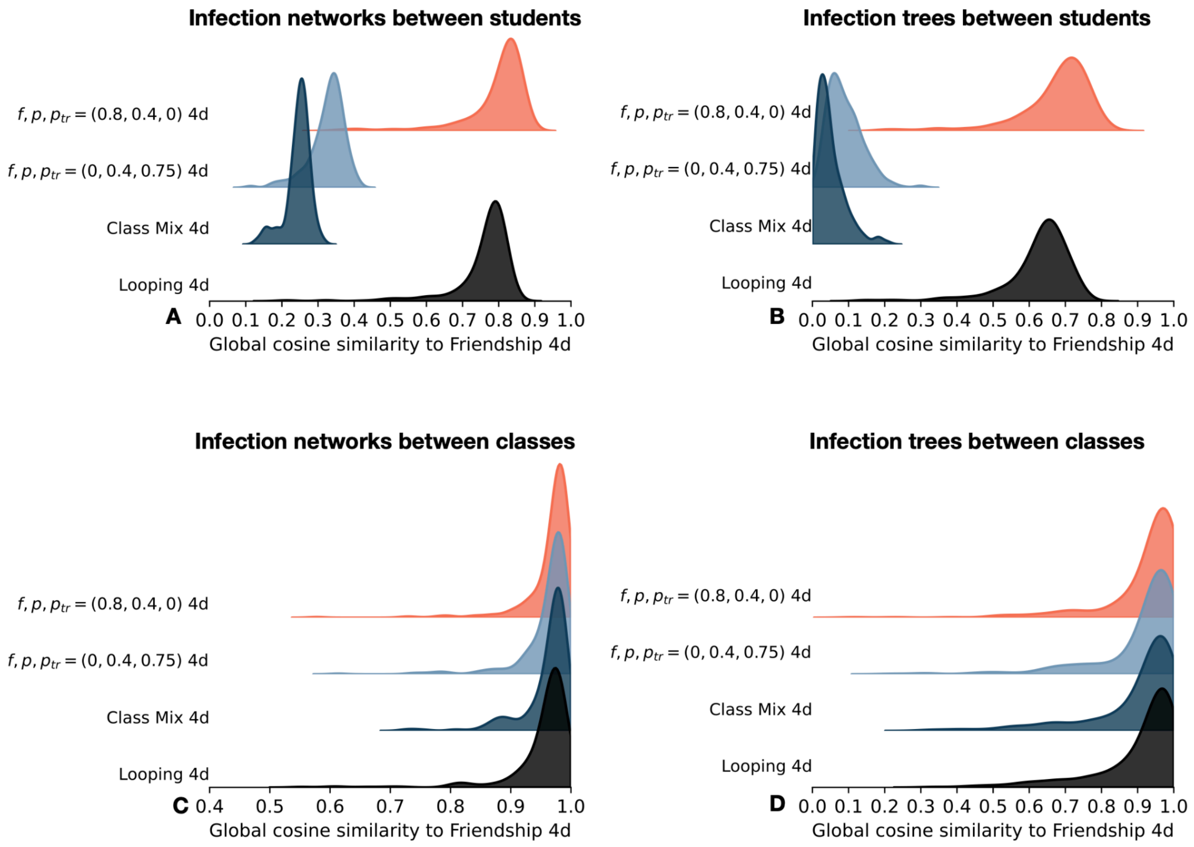

Figure 11: *Infection pathways obtained from the ``Friendship 4d" contact sequence are compared to those obtained with contacts generated with the friendship-based algorithm with parameter triplets f= 0.8, p =0.4 and p_tr = 0 (transitivity mechanism turned off), f= 0, p =0.4 and p_tr = 0.75 (friendship mechanism turned off). Comparison with ``Looping 4d" and ``Class Mixing 4d" are reported for reference. Panel A displays the distributions (Gaussian kernel density estimations) over all seeds of the global cosine similarities GCS(G_inf(s, ct_a), G_inf(s, ct_b)) between infection networks between students. Panel B instead shows the distributions of the*

global cosine similarities between infection trees instead. The bottom row instead compares infection networks (panel C) and infection trees (panel D) between classes. For each contact sequence, infection networks are obtained from 150 simulations for each seed, and each of the 325 students are successively considered as seed s.

Minor Comments

1. As most of the box plots in Figure 5, Panels E and F are fully above a cosine similarity of 0.8, it would be easier to see the differences between different synthetic data generation mechanisms, if the y-axis limits were adapted to the range 0.75 – 1.0 or if a zoom into this region would be provided as an inset.

We thank the Reviewer for their suggestion. In order to improve the readability of Figure 5 we have now opted for a different style of visualisation that can be applied on all panels as suggested by Reviewer #3 (point 3). In the resubmitted manuscript, all panels in Figure 5 now display the entire distributions (instead of boxplots) and therefore extend on a wider range of values of the global cosine similarity. We have nonetheless adjusted the limits of the relevant axes where possible in order to frame the data closely. Figure 5 has now been replaced with the alternative shown below.

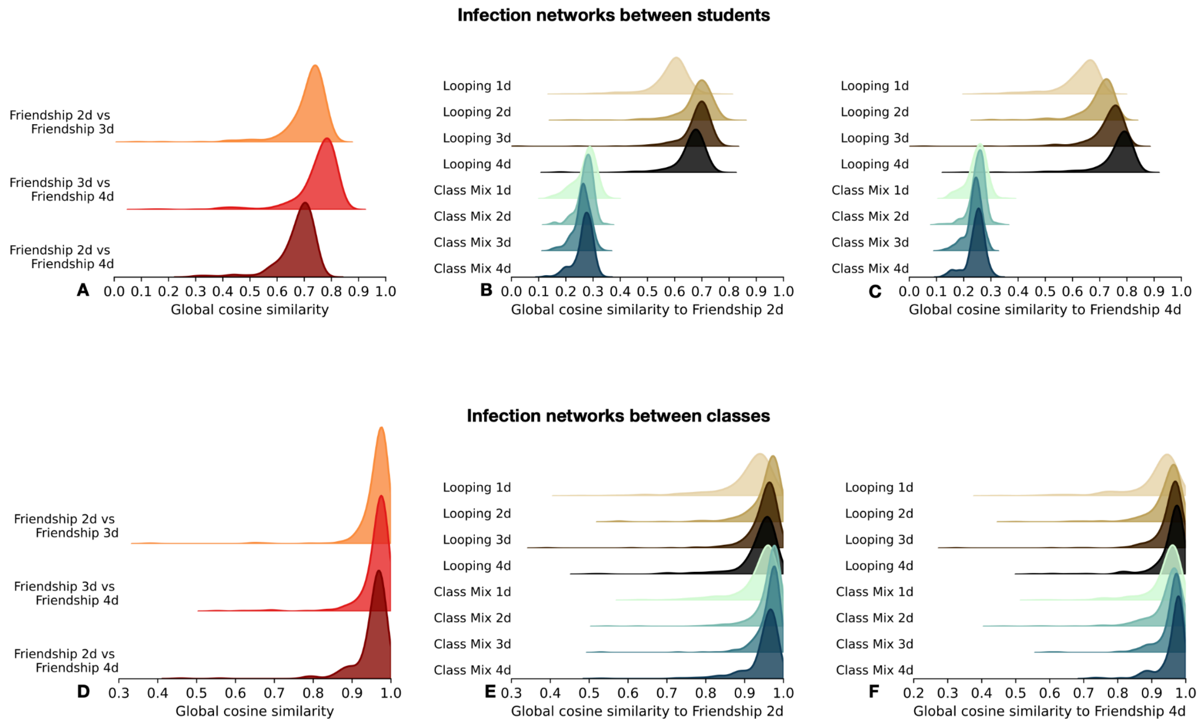

Figure 5: *Distributions (Gaussian kernel density estimations) over all seeds of the global cosine similarities GCS(G_inf(s, ct_a), G_inf(s, ct_b)) are shown for infection networks obtained from pairs of contact sequences ct_a and ct_b in ``Friendship 2d'', ``Friendship 3d'' and "Friendship 4d'' in panels A (for infection networks between students) and D (for infection networks between classes). In panels B (students) and E (classes), ct_a is fixed to ``Friendship 2d'', and the distribution of GCS(G_inf(s, ct_a), G_inf(s, ct_b)) over all seeds s is shown for different sequences ct_b (class-mixing-based and looped contacts); this procedure is repeated in panels*

C (between students) and F (between classes) with ct_a=``Friendship 4d''. For each contact sequence, infection networks are obtained from 150 simulations for each seed, and each of the 325 students are successively considered as seed s.

Reviewer #3

The manuscript describes a method to synthesize contact patterns by temporally extending empirical data from schools. Their method accounts for repeated contacts by distinguishing between friendship and casual contacts. They also propose a method that only preserves the class-mixing patterns. The authors argue that their synthetic contacts preserve multiple features of the empirical dataset, that individual infection pathways differ between their methods but between-class pathways are similar, and that using 2 days of data produces similar outcomes as of using 3 or 4 days.

The manuscript is overall clear and well written, and the methods look scientifically sound. The authors show awareness of the surrounding literature, fitting their work into a relevant gap. Their method can be a great tool for studying local outbreaks in school settings. Worth mentioning, the GitHub repo with the code and data is very organized and easy to navigate. I comment below about a few issues, which I consider to be minor.
We thank the Reviewer for their positive feedback and comments on our manuscript. We are particularly pleased that the GitHub repo was easy to use, and hope that this promotes further work on designing plausible contact patterns for applied infectious disease modelling. We are grateful for the few issues raised by the Reviewer on our manuscript, as well as their suggestion to resolve them. We have followed all of them, and hope that the Reviewer finds our manuscript improved.

1. Ideally, it would be great to validate the proposed model with a dataset that spans for a longer time frame – say, as long as a typical outbreak would last for. By this, I mean to check for how long the synthetic contacts still reproduce the features of the dataset, as friendships and casual contacts evolve over time in schools. I understand that this requires a long-term dataset, spanning time frames longer than a week. Is such a dataset available? If so, using this to validate the model would be an important future step, and the authors could mention this in discussion. Otherwise, adding this limitation to the paper's discussion could be a push for the collection of longer-term contact patterns in schools. I understand that this issue is beyond scope and does not impair the worthiness of the manuscript.

The Reviewer raises an excellent point. Indeed, such a data set would provide further insight on the performance of the friendship-based algorithm.

Existing data sets that cover sufficient time scales to be used as suggested by the Reviewer are not suitable. The Copenhagen Network Study (Sapiezynski et al, Scientific Data 2019) provides proximity contacts captured using mobile phone bluetooth sensing among university students. Resulting contacts are captured with a wider detection range (up to 10m), and are not exclusively face-to-face. The i-bird study (Obadia et al, PLOS Computational Biology 2015) instead captured long-term face-to-face contacts in a healthcare facility using the same technology as the school contacts used in our manuscript. However, the different context makes it unsuitable for the purpose of validating our algorithm further.

As the Referee suggests, acquiring such a data set in schools would be highly valuable for future research. We have now mentioned this in the limitations of the resubmitted manuscript with the following text.

> *Finally, further validation of our algorithm is limited by the absence of longer recordings gathered in the school setting. Acquiring such datasets would be highly valuable for future research.*

2. Figure 4.D). While I understood the main message from panels A, B and C, panel D was more difficult for me to read. Checking the bubbles and their sizes was rather distracting, so can the authors consider an alternative visualization? Since the idea is to compare different distributions in pairs, maybe a triangular matrix would be more effective (this is, each row and column represents one approach, and the matrix entry, which could be color-coded, represents the Jensen-Shannon distance). Another possibility would be to use bars instead of the bubbles.

We thank the Reviewer for highlighting this and suggesting alternative visualisations. We have now modified panel D of figure 4, in order to use a triangular matrix to display the Jensen-Shannon distance, as suggested by the Reviewer. The resubmitted version of Figure 4 is shown below.

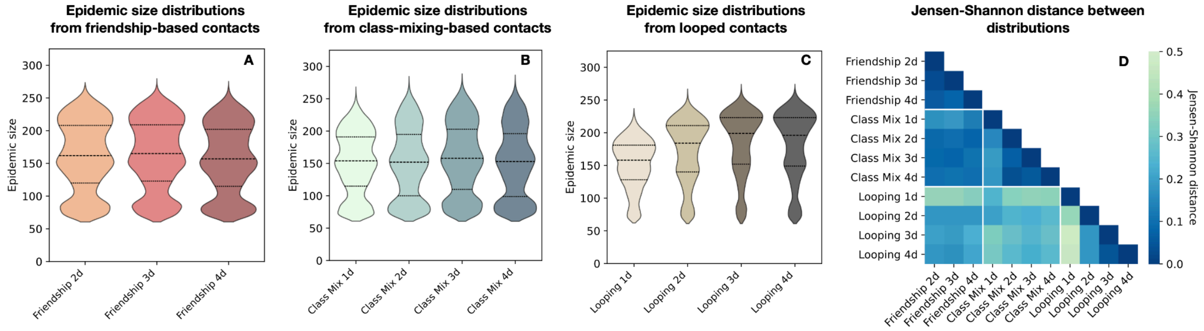

Figure 4: *Distributions (Gaussian kernel density estimations) of the final epidemic sizes obtained with friendship-based, class-mixing-based and looped contact sequences are shown in Panels A, B and C. The distributions are computed over simulations leading to a fraction of infected individuals larger than 20% (over 120 days) in order to better highlight differences between the distributions. Results including all simulations are shown in the Supplementary Information, Fig. 12. The first and third quartiles (25% and 75%) are indicated with dotted lines while the median is shown with a dashed line. The Jensen-Shannon distance between each pair of distributions is shown in Panel D. For each contact sequence, 150 simulations are conducted for each of the 325 students as seed (48,750 simulations for each contact sequence).*

All Figures in the Supplementary Information comparing the epidemic size distributions have also been edited in order to uniformise the presentation, by replacing the previous visual style (scatter plot) with a triangular matrix.

3. Figure 5. While all panels in Fig. 5 represent the same quantity (cosine similarity between infection networks), the multiple plot styles employed made it difficult for me to decipher the message. I believe that using a single plot style would be better, so the reader does

not have to learn how to read the data in different ways, making the main message clearer. I particularly prefer the filled curve style of panels B and C.

We thank the reviewer for their suggestion. Indeed, a single plot style is best for readability. In this figure, the different distributions in panels D-E-F overlap to the extent that the style used in panel B and C does not result in easily read panels E and F (see figure below). This originally motivated our use of different styles.

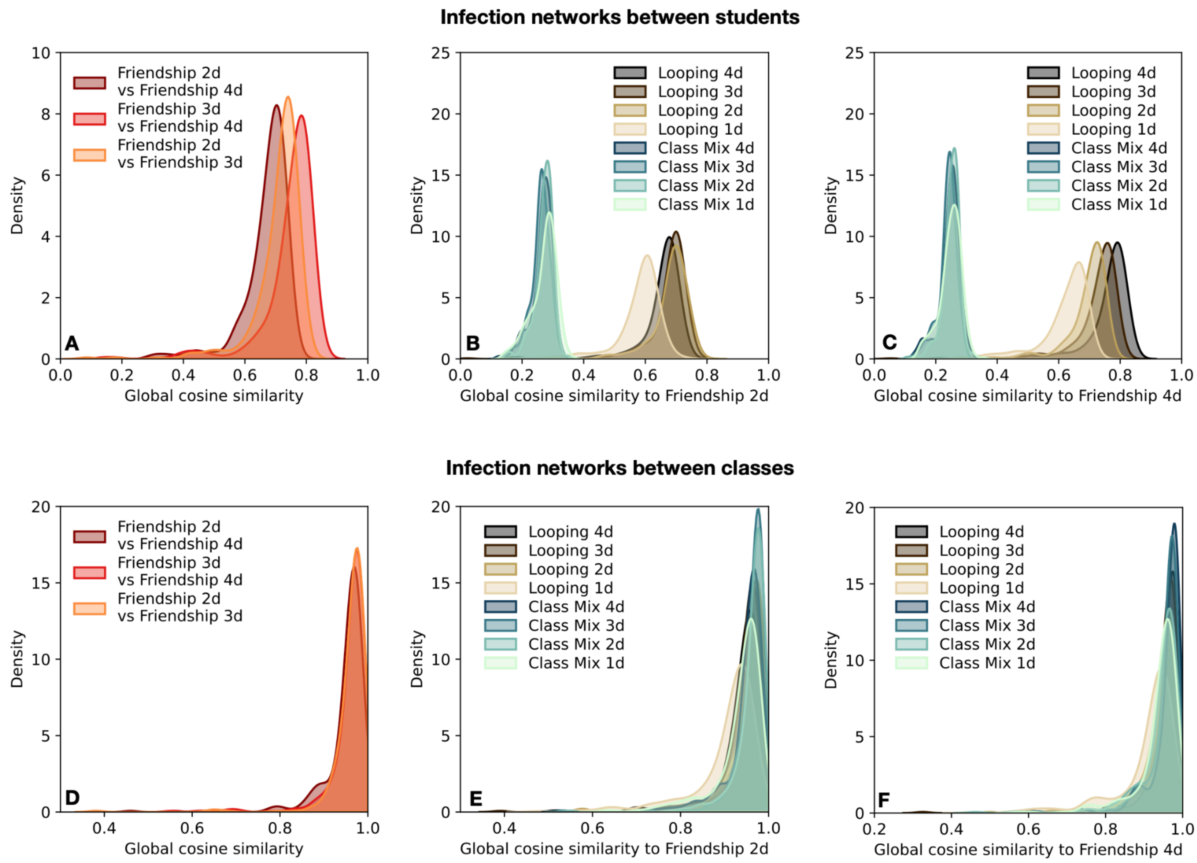

Distributions (Gaussian kernel density estimations) over all seeds of the global cosine similarities GCS(G_inf(s, ct_a), G_inf(s, ct_b)) are shown for infection networks obtained from pairs of contact sequences ct_a and ct_b in ``Friendship 2d'', ``Friendship 3d'' and "Friendship 4d" in panels A (for infection networks between students) and D (for infection networks between classes). In panels B (students) and E (classes), ct_a is fixed to ``Friendship 2d'', and the distribution of GCS(G_inf(s, ct_a), G_inf(s, ct_b)) over all seeds s is shown for different sequences ct_b (class-mixing-based and looped contacts); this procedure is repeated in panels C (between students) and F (between classes) with ct_a=``Friendship 4d''. For each contact sequence, infection networks are obtained from 150 simulations for each seed, and each of the 325 students are successively considered as seed s.

In order to uniformise the styles across all panels, we have opted instead for an alternative style that we believe allows each panel to remain legible. We hope that the Reviewer finds it improved. The resubmitted version of Figure 5 is shown below.

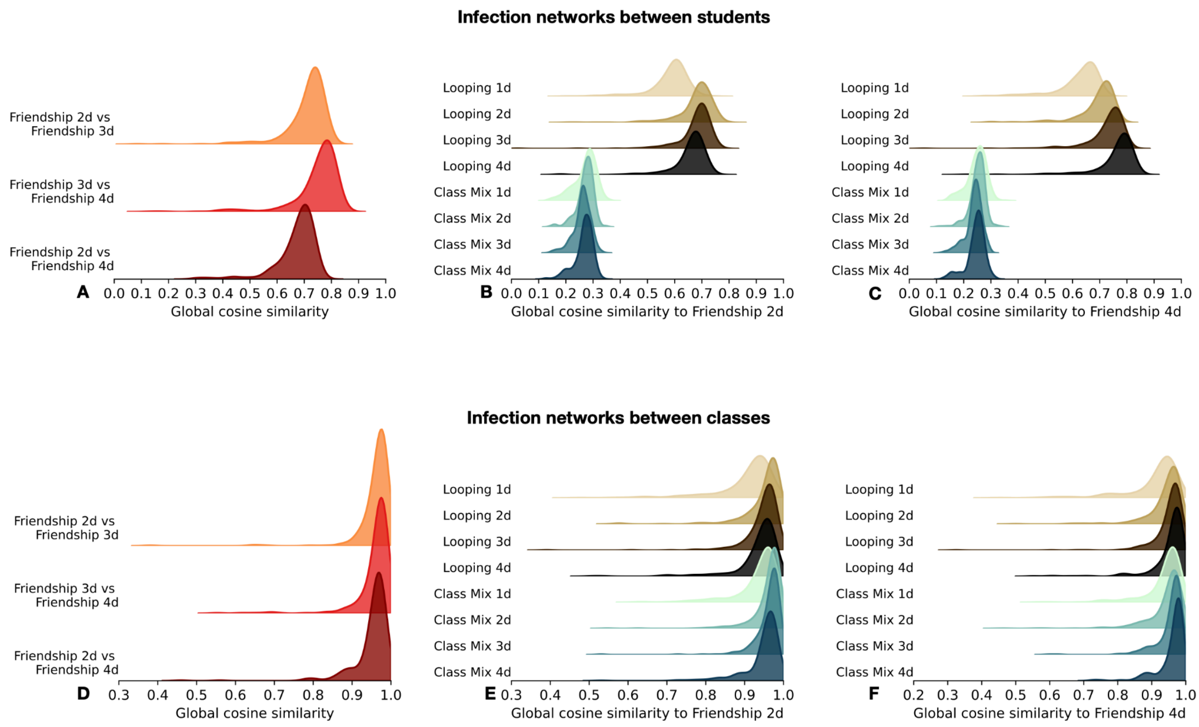

Figure 5*: Distributions (Gaussian kernel density estimations) over all seeds of the global cosine similarities GCS(G_inf(s, ct_a), G_inf(s, ct_b)) are shown for infection networks obtained from pairs of contact sequences ct_a and ct_b in ``Friendship 2d'', ``Friendship 3d'' and "Friendship 4d'' in panels A (for infection networks between students) and D (for infection networks between classes). In panels B (students) and E (classes), ct_a is fixed to ``Friendship 2d'', and the distribution of GCS(G_inf(s, ct_a), G_inf(s, ct_b)) over all seeds s is shown for different sequences ct_b (class-mixing-based and looped contacts); this procedure is repeated in panels C (between students) and F (between classes) with ct_a=``Friendship 4d''. For each contact sequence, infection networks are obtained from 150 simulations for each seed, and each of the 325 students are successively considered as seed s.*

All figures comparing infection pathways in the Supplementary Information have also been edited to unify the style of presentation.

4. On line 108: I guess the word "distributions" is repeated.

We thank the Reviewer for pointing this out, this typo has been corrected.

5. On line 286: "when a susceptible individual is in contact with an infection one" – perhaps you meant "infectious".

We thank the Reviewer for pointing this out, this typo has been corrected.
